# A novel solution to optimal power flow problems using composite differential evolution integrating effective constrained handling techniques

**DOI:** 10.1038/s41598-024-56590-5

**Published:** 2024-03-14

**Authors:** Aamir Ali, Ali Hassan, M. U. Keerio, Noor H. Mugheri, Ghulam Abbas, Mohammed Hatatah, Ezzeddine Touti, Amr Yousef

**Affiliations:** 1 Department of Electrical Engineering, Quaid-e-Awam University of Engineering Science and Technology, Nawabshah, Sindh 67450 Pakistan; 2 School of Electrical Engineering, Southeast University, Nanjing, 210096 China; 3 Department of Electrical Engineering, Al-Baha University, 65779-7738 Alaqiq, Saudi Arabia; 4 Department of Electrical Engineering, College of Engineering, Northern Border University, Arar, 91431, Saudi Arabia; 5 Department of Electrical Engineering, College of Engineering, University of Business and Technology, 21589 Jeddah, Saudi Arabia; 6 Engineering Mathematics Department, Faculty of Engineering, Alexandria University, Lotfy El-Sied st. off Gamal Abd El-Naser Alexandria, 11432, Egypt

**Keywords:** Power loss and emission, Optimal power flow, Constraint handling techniques, Feasibility rule, ε Constrained method, Constrained composite differential evolution, Engineering, Energy science and technology, Energy infrastructure, Renewable energy

## Abstract

Optimal power flow is a complex and highly non-linear problem in which steady-state parameters are needed to find a network’s efficient and economical operation. In addition, the difficulty of the Optimal power flow problem becomes enlarged when new constraints are added, and it is also a challenging task for the power system operator to solve the constrained Optimal power flow problems efficiently. Therefore, this paper presents a constrained composite differential evolution optimization algorithm to search for the optimum solution to Optimal power flow problems. In the last few decades, numerous evolutionary algorithm implementations have emerged due to their superiority in solving Optimal power flow problems while considering various objectives such as cost, emission, power loss, etc. evolutionary algorithms effectively explore the solution space unconstrainedly, often employing the static penalty function approach to address the constraints and find solutions for constrained Optimal power flow problems. It is a drawback that combining evolutionary algorithms and the penalty function approach requires several penalty parameters to search the feasible space and discard the infeasible solutions. The proposed a constrained composite differential evolution algorithm combines two effective constraint handling techniques, such as feasibility rule and ɛ constraint methods, to search in the feasible space. The proposed approaches are recognized on IEEE 30, 57, and 118-bus standard test systems considering 16 study events of single and multi-objective optimization functions. Ultimately, simulation results are examined and compared with the many recently published techniques of Optimal power flow solutions owing to show the usefulness and performance of the proposed a constrained composite differential evolution algorithm.

## Introduction

The optimal power flow (OPF) integrates the computation of power flow and economic dispatch subject to the system’s physical and electrical constraints^1^. In the research field of electrical power systems, OPF is an extensively sophisticated topic due to various interesting challenges, and it possesses both the planning and operating stages. In OPF, perfect values of control variables and system quantities are calculated to find the most efficient system operation and planning subject to various constraints. Many classical mathematical techniques have succeeded in finding the solution to the OPF problem, including the Newton method, linear, non-linear, quadratic programming, and interior point method. These techniques are limited to handling algebraic functions only. They cannot consider the convexity, require initial point, more significant control parameters, and continuity assumptions, and are gradient-based search algorithms trapped into local optima^2^.

In the past few years, numerous metaheuristic algorithms have been introduced to find better results for OPF problems, and most of these methods successively overcome the limitations of classical techniques that not only stagnate into local optima but are also unable to explore the global optima. These algorithms include a differential search algorithm (DSA)^3^ proposed by Abaci and Yamacli, who considered various single and multi-objective functions to optimize standard IEEE systems, in^4^ adaptive group search optimization (AGSO) proposed by Daryani et al. to solve OPF problem considering multi-objective function model, backtracking search optimization algorithm (BSA) in^5^ wherein valve-point loading and multi-fuel cost are considered for the output of thermal power generators. Furthermore, differential evolution (DE) with the integration of various constraint techniques^6^, multi-objective differential evaluation algorithm (MO-DEA)^7^, moth swarm algorithm (MSA)^8^, improved colliding bodies optimization (ICBO)^9^, chaotic artificial bee colony (CABC)^10^, Gbest ABC (GABC)^11^, adaptive real coded biography based optimization algorithm (ARCBBO) was suggested in^12^, adaptive partitioning flower pollination algorithm (APFPA)^13^ was used to resolve OPF problems considering various single and multi-objective objective functions. Pandiarajan and Babulal^14^ proposed the integration of a fuzzy and harmony search algorithm (HSA) called (FHSA) to figure out the OPF problem; by doing this, two HSA parameters (i-e. bandwidth and pitch adjustment) were controlled by the fuzzy logic system. Furthermore, a combination of lévy mutation and teaching learning-based optimization (LTLBO) technique proposed in^15^, krill herd algorithm (KHA) in^16^ and stud KHA (SKHA) in^17^, glowworm swarm optimization^18^, hybrid modified imperialist competitive algorithm (MICA) and teaching–learning algorithm (TLA) (MICA-TLA)^19^ there has also popular optimization techniques for searching the OPF problem solution. However, Objectives in OPF problems are variable, where no single algorithm is the best to address every objective function of OPF problems. Therefore, there is room for the new algorithm to solve most of the OPF problems efficiently.

This paper proposed optimizing single and Multi-objective approaches to solving OPF problems. Existing work in the literature clearly shows that the basic or improved version of optimization algorithms was used to solve the OPF problems. Each method has its strong points and limitations, and it is confirmed in the No Free Lunch (NLF) theorem^20^, which indicates that no single optimization algorithm can solve in the best way for all types of real word problems. Recently, an outstanding global optimizer-constrained composite differential evolution (C2oDE)^21^ algorithm has had various advantages, i.e., Simple in structure, implemented easily in any programming language, with few control parameters, combining the strength of different trial vector generation strategies.

Furthermore, to handle the constraints of OPF problems, mainly in the entire literature, researchers either adopt the static penalty function or directly discard the infeasible population. The former method is more responsive to selecting the penalty coefficient; even if a small penalty coefficient may cause examination of the infeasible space, a significant coefficient of penalty function may not explore the entire search space. However, in the OPF problem, recent advanced constraint handling techniques (CHTs) still need to be used. Therefore, in this paper, feasibility rule (FR), ɛ-constraint method (ECM), and a combination of these CHTs are utilized to solve the OPF problem by employing a composite DE search algorithm. Moreover, the performance of each CHTs and their varieties, such as C2oDE-FR, C2oDE-ECM, C2oDE-FR-ECM, and C2oDE-ECM-FR, have been statistically analyzed and compared. Besides, proposed CHTs are implemented successfully to solve the OPF problem on a small scale IEEE 30, 57 and a large-scale power network of 118-bus test systems. Most objective functions from the literature review, such as cost of active power generation, emission rate of greenhouse gases, power loss, voltage deviation, and voltage stability index, are considered to test the performance of the proposed C2oDE algorithm along with the integration of CHTs. Correspondingly, sixteen events of single and Multi-objective functions are formulated to test the efficacy of various CHTs. The simulation results of all events are thoroughly examined and compared with the latest research findings.

The contributions of the study are outlined as follows:

- Two representative constraint techniques, such as feasibility rule (FR) and epsilon constraint method (ECM), and their combinations are employed with the current state-of-the-art unconstrained CoDE search algorithm to solve the OPF problem.
- Sixteen events of highly complex non-linear objective functions are formulated to solve single and multi-objective OPF problems and show the superiority and performance of the proposed algorithm.
- Simulation results of all the algorithms C2oDE-FR, C2oDE-ECM, C2oDE-FR-ECM, and C2oDE-ECM-FR are statistically compared.
- Small to large-scale power system networks such as IEEE 30, 57, and 118-bus networks are adopted to test the proposed Algorithm.

The remaining division of this article is planned as Sect. 2 contains mathematical modeling of OPF and constraint handling techniques, and Sect. 3 describes the objective function and study events. The proposed optimization algorithm is defined in Sect. 4, simulation results and comparisons are discussed in Sect. 5, and concluding remarks are produced in Sect. 6.

## Mathematical modelling of OPF problem

Generally, OPF is a complex and non-linear problem, and its main objective is to optimize single and multi-objective functions subject to satisfy the set of equality and inequality constraints. Mathematically, the OPF problem is described as follows:

Minimize $$f\left(\overrightarrow{x},\overrightarrow{u}\right), \overrightarrow{x} \wedge \overrightarrow{u}\in S, {L}\le \left(\overrightarrow{x},{\overrightarrow{u}}\right)\le {U}$$

$$\mathrm{Subject to}: {g}_{j}\left(\overrightarrow{x},\overrightarrow{u}\right)\le 0,\quad j=1,\dots , l$$

1 $${h}_{j}(\overrightarrow{x},\overrightarrow{u})=0,\quad j=l+1, \dots , m$$

whereas $f\left(\overrightarrow{x},\overrightarrow{u}\right)$ is the fitness function, ${g}_{j}\left(\overrightarrow{x},\overrightarrow{u}\right)$ and ${h}_{j}(\overrightarrow{x},\overrightarrow{u})$ are the inequality and equality constraints, vector $\overrightarrow{x}$ are dependent or state variables, $\overrightarrow{u}$ is independent or control variables. *S* is the search space, *L* and *U* are the lower and upper bound, r espectively of vectors $\overrightarrow{x}$ and $\overrightarrow{u}$.

### State and control variables

**Figure 1:**
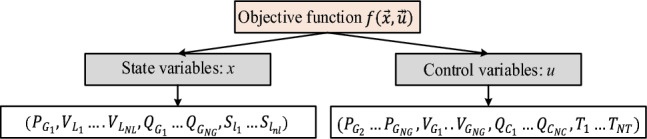
State and control variables.

The state variables describe the power system's state, and the power flow in the network is controlled by control variables shown in Fig. 1. Where, *NG*, *NL*, *NC,* and *NT* are the number of generators, load, shunt VAR compensator, and transformer buses respectively and *nl* shows the number of branches.

### Constraints and constraint handling techniques

#### Constraints

**Figure 2:**
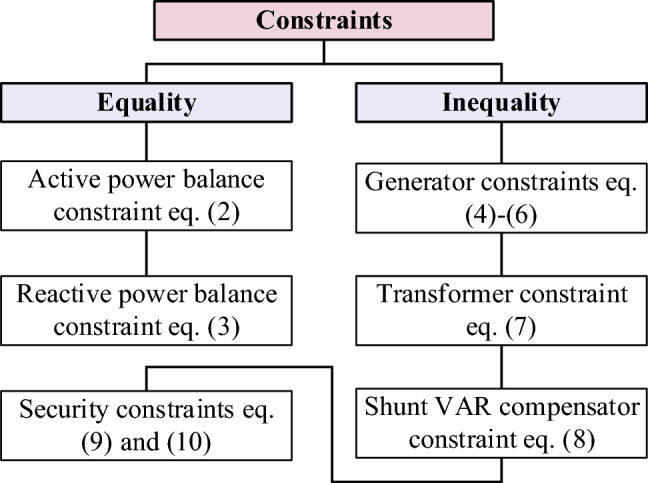
Equality and inequality constraints.

The solution to the OPF problem must achieve both equality (active and reactive power balance) and inequality (operating limits of power system components) constraints. Figure 2 shows the equality and inequality constraints examined in the present study.

2 $${P}_{{G}_{i}}-{P}_{{D}_{i}}-{V}_{i}\sum_{j=1}^{NB}{V}_{j}\left[{G}_{ij}\mathrm{ cos }\left({\delta }_{ij}\right)+{B}_{ij}\mathrm{ sin }\left({\delta }_{ij}\right)\right]=0\, \forall i\in NB$$

3 $${Q}_{{G}_{i}}-{Q}_{{D}_{i}}-{V}_{i}\sum_{j=1}^{NB}{V}_{j}\left[{G}_{ij}\mathrm{ sin }\left({\delta }_{ij}\right)-{B}_{ij}\mathrm{ cos }\left({\delta }_{ij}\right)\right]=0\, \forall i\in NB$$

where, ${P}_{{D}_{i}}$ and ${Q}_{{D}_{i}}$ are the active and reactive demand at bus *i*, ${G}_{ij}$ and ${B}_{ij}$ are shunt conductance and susceptance between bus *i* and *j* respectively.${\delta }_{ij}$ is the voltage angle difference between bus *i* and *j* and shows *NB* the number of buses.

4 $${V}_{{G}_{i}}^{ min }\le {V}_{{G}_{i}}\le {V}_{{G}_{i}}^{ max } \,\forall i\in NG$$

5 $${P}_{{G}_{i}}^{\mathrm{ min }}\le {P}_{{G}_{i}}\le {P}_{{G}_{i}}^{\mathrm{ max }} \,\forall i\in NG$$

6 $${Q}_{{G}_{i}}^{\mathrm{ min }}\le {Q}_{{G}_{i}}\le {Q}_{{G}_{i}}^{\mathrm{ max }} \,\forall i\in NG$$

7 $${T}_{j}^{ min }\le {T}_{j}\le {T}_{j}^{ max }\, \forall j\in NT$$

8 $${Q}_{{C}_{k}}^{ min }\le {Q}_{{C}_{k}}\le {Q}_{{C}_{k}}^{ max }\, \forall k\in NC$$

9 $${V}_{{L}_{p}}^{ min }\le {V}_{{L}_{p}}\le {V}_{{L}_{p}}^{ max }\, \forall p\in NL$$

10 $${S}_{{l}_{q}}\le {S}_{{l}_{q}}^{ max }\, \forall q\in nl$$

At the time of the optimization process, the proposed algorithm chooses the values of each variable between the min and max limit.

#### Proposed constraint handling techniques

Usually, all the real word problems are constraint type defined in Eq. (1), in which the equality constraints ${h}_{j}(\overrightarrow{x},\overrightarrow{u})$ given in Eqs. (2) and (3) are automatically satisfied when the solution of power flow is converged. However, special attention is needed to inequality constraints ${g}_{j}\left(\overrightarrow{x},\overrightarrow{u}\right)$ given in Eqs. (4) to (10). Generally, the *j*^*th*^ inequality constraint violation ${G}_{j}(\overrightarrow{x})$ is given as:

11 $${G}_{j}\left(\overrightarrow{x}\right)={\text{max}}\left(0,{g}_{j}\left(\overrightarrow{x},\overrightarrow{u}\right)\right), 1\le j\le l$$

However, the overall degree of constraint violation $G(\overrightarrow{x})$ can be calculated by the sum of all the inequality constraint violations and given as:

12 $$G(\overrightarrow{x})={\sum }_{j=1}^{m}{G}_{j}(\overrightarrow{x})$$

Constrained optimization problems mean to search in the feasible region, and EAs are population-based stochastic search methods in which an infeasible solution is complicated to discard. Therefore, proper CHTs are used together with EAs to enhance the overall performance of an algorithm. This work proposes two CHTs: feasibility rule (FR) and ε constrained method (ECM). FR is given in^22^ and suggests three rules to compare any two solutions described as follows:

1. Both solutions are feasible; select the one with a better objective function value.
2. Both solutions are infeasible; choose the one with a lower value of constraint violation.
3. One is feasible, and the other is infeasible; always select a feasible one.

The second proposed CHT is ECM has given in^23,24^, in which two solutions ${\overrightarrow{x}}_{i}$ and ${\overrightarrow{x}}_{j}$ are compared as follows:

13 $$\left\{ {\begin{array}{*{20}l} {f\left( {\vec{x}_{i}^{{\text{~}}} } \right) < f\left( {\vec{x}_{j}^{{\text{~}}} } \right),} & {if{\text{~}}G\left( {\vec{x}_{i}^{{\text{~}}} } \right) < \varepsilon \wedge G\left( {\vec{x}_{j}^{{\text{~}}} } \right) \le \varepsilon } \\ {f\left( {\vec{x}_{i}^{{\text{~}}} } \right) < f\left( {\vec{x}_{j}^{{\text{~}}} } \right),} & {{\text{~}}if{\text{~}}G\left( {\vec{x}_{i}^{{\text{~}}} } \right) = G\left( {\vec{x}_{j}^{{\text{~}}} } \right)} \\ {G\left( {\vec{x}_{i}^{{\text{~}}} } \right) < G\left( {\vec{x}_{j}^{{\text{~}}} } \right),} & {otherwise} \\ \end{array} } \right.$$

In (13), the parameter $\varepsilon$ decays as increasing the iteration number and is given as

14 $$\varepsilon =\left\{\begin{array}{ll}{\varepsilon }_{0}(1-\frac{t}{T}{)}^{cp},& if \frac{t}{T}\le p\\ 0,& otherwise\end{array}\right.$$

15 $$cp=-\frac{\mathrm{ log }{\varepsilon }_{0}+\lambda }{\mathrm{ log }(1-p)}$$

where the parameter *ε*_0_ is the primary threshold, initially it is equal to $max(G\left(\overrightarrow{x}\right))$, *T* is the maximum generation, *t* is the current generation, constant parameter *λ* = 6 recommended in^25^ and *p* controls the degree of convergence of objective function.

## Objective functions and study events

To highlight the superiority and effectiveness of the proposed C2oDE algorithm by considering the various CHTs, 16 events comprised of single and multi-objective functions are evaluated and implemented on IEEE 30, 57, and 118-bus standard IEEE networks. Bus 1 is considered the slack/reference bus in the event of 30 and 57-bus systems; however, in the 118-bus system, the 69th bus is the slack/reference bus. The role of the reference bus is to achieve equality constraints given in Eqs. (2) and (3) during the load flow study. In subsequent sub-sections, the mathematical formulation of different events for the 30, 57, and 118-bus tests is described.

### IEEE 30-bus system

The base MVA, bus, branch, and generator data of the IEEE 30-bus test network is taken from^26^, and a summary of the significant components of this system is arranged in Table 1. There are 10 events are formulated for the IEEE 30-bus network, in which the first six events comprised of minimizing single objective and the remaining four events are based on weighted sum multi-objective optimization.

**Table 1:** Summary of IEEE 30-bus test system under study.

Items	Quantity	Details
Buses (slack Bus)	30 (1)	^27^
Generator buses	06	1, 2, 5, 8, 11, 13
Independent variable buses	24	–
Shunt VAR compensator buses	9	10, 12, 15, 17, 20, 21, 23, 24, 29
Total active and reactive demand	–	283.4 MW, 126.2 MVAr
Branches	41	^26^
Tap changer transformer branches	4	11, 12, 15, 36
Voltage range at slack and PV buses	5	[0.95–1.1] p.u
Voltage range at PQ buses	24	[0.95–1.05] p.u

#### Event 1: minimization of basic fuel cost

Almost in all the literature, minimization of fuel cost was considered, and the relationship between the generator output power (MW) and the fuel cost ($/h) is given by a quadratic curve described as:

16 $$f(x, u)={\sum }_{i=1}^{NG}{a}_{i}+{b}_{i}{P}_{{G}_{i}}+{c}_{i}{P}_{{G}_{i}}^{2}$$

where, ${P}_{{G}_{i}}$ is the generated output power of *i*^*th*^ bus and ${a}_{i}, {b}_{i}, {c}_{i}$ The constant cost coefficients of that generator are given in^5,27^ and classified as in Table 2.

**Table 2:** Coefficients of cost and emission (generators) for 30-bus network.

Generator	Bus	a	b	c	d	e	$\alpha$	$\beta$	$\gamma$	$\omega$	$\mu$
*G*_*1*_	1	0	2	0.00375	18	0.037	4.091	− 5.554	6.49	0.0002	2.857
*G*_*2*_	2	0	1.75	0.0175	16	0.038	2.543	− 6.047	5.638	0.0005	3.333
*G*_*3*_	5	0	1	0.0625	14	0.04	4.258	− 5.094	4.586	0.000001	8
*G*_*4*_	8	0	3.25	0.00834	12	0.045	5.326	− 3.55	3.38	0.002	2
*G*_*5*_	11	0	3	0.025	13	0.042	4.258	− 5.094	4.586	0.000001	8
*G*_*6*_	13	0	3	0.025	13.5	0.041	6.131	− 5.555	5.151	0.00001	6.667

#### Event 2. minimization of fuel cost multi-fuels

**Figure 3:**
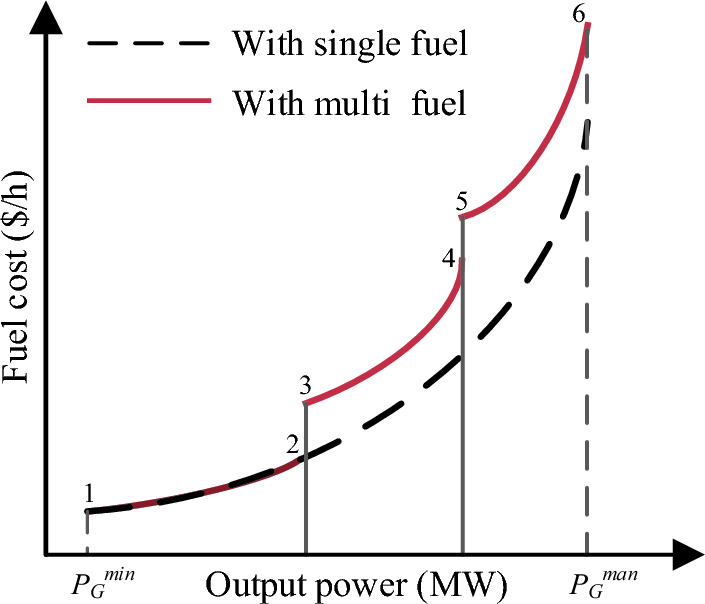
Output power vs fuel cost of single and multi-fuels.

Thermal power generation may have multi-fuel resources, including coal, oil, and natural gas. Therefore, the relationship between fuel cost vs output power for such plants is given in the piecewise quadratic function shown in Fig. 3.

Mathematically, the cost function of a multi-fuel *i*^*th*^ generator is given as follows:

17 $${f}_{i}\left(x, u\right)={a}_{ik}+{b}_{ik}{P}_{{G}_{j}}+{c}_{ik}{P}_{{G}_{i}}^{2}\,\mathrm{ for }\,fue{l}_{k}$$

where, ${P}_{{G}_{i}}$ is the generator output power within the specified range of $\left[{P}_{{G}_{ik}}^{\mathrm{ min }}, {P}_{{G}_{ik}}^{\mathrm{ max}}\right]$ and k is the fuel type. The total fuel cost of the objective function can be calculated using Eq. (18).

18 $$f(x, u)=\left({\sum }_{i=1}^{NG}{f}_{i}(x, u)\right)$$

In this event, the multi-fuel cost is proposed for the two generators and range of output power (MW) with their coefficients given in^5^ and shown in Table 3, whereas, the cost for the other four generators is identical as in the event 1.

**Table 3:** Multi-fuel cost coefficients of generators 1 and 2 of the IEEE 30-bus test system.

Generator_Bus_	*P*_*min*_–*P*_*max*_	a	b	c
*G*_*1*_	50–140	55	0.7	0.005
	140–200	82.5	1.05	0.0075
*G*_*2*_	20–55	40	0.3	0.01
	55–80	80	0.6	0.02

#### Event 3: voltage stability improvement

Estimate of voltage stability is an issue that is receiving growing attention from power system researchers due to system collapses in the past because of voltage instability. Voltage stability index (*L*_*max*_) has developed which can be defined based on *L*_*j*_ local indicator. Let *NG* and *NL* be the number of generator and load buses respectively, and then local indicator *L*_*j*_ can be calculated as

$${L}_{j}=\left|1-{\sum }_{i=1}^{NG}{F}_{ji}\frac{{V}_{i}}{{V}_{j}}\right|,\, where\, j=1, 2,\dots ,NL$$

19 $$and\, {F}_{ji}=-[{Y}_{LL}{]}^{-1}[{Y}_{LG}]$$

where sub-matrices ${Y}_{LL}$ and ${Y}_{LG}$ are calculated from the *Y*_*BUS*_ matrix after separating PV and PQ buses as given in (17).

20 $$\left[\begin{array}{l}{I}_{L}\\ {I}_{G}\end{array}\right]=\left[\begin{array}{ll}{Y}_{LL}& {Y}_{LG}\\ {Y}_{GL}& {Y}_{GL}\end{array}\right]\left[\begin{array}{l}{Y}_{L}\\ {Y}_{G}\end{array}\right]$$

The objective function of power system stability in this event is the maximum value of *L*_*j*_ and is given as:

21 $$f(x, u)={L}_{max}=\mathrm{ max }({L}_{j}),\, where\, j=\mathrm{1,2}, \dots , NL$$

#### Event 4: minimization of emission

Many harmful gases such as SO_x_ and NO_x_ are emitted in tones per hour (t/h) into the atmosphere using conventional fuel's thermal power generation (MW). In the present event, the emission is considered the objective function of OPF and computed as:

22 $$\mathrm{Emission }={\sum }_{i=1}^{NG}[({\alpha }_{i}+{\beta }_{i}{P}_{{G}_{i}}+{\gamma }_{i}{P}_{{G}_{i}}^{2})\times 0.01+{\omega }_{i}{e}^{({\mu }_{i}{P}_{{G}_{i}})}]$$

where, the values of the parameters ${\alpha }_{i}, {\beta }_{i}, {\gamma }_{i}, {\omega }_{i}$ and ${\mu }_{i}$ are given in Table 2.

#### Event 5: active power loss minimization

Mathematically active power loss (MW) can be given as:

23 $${P}_{loss}={\sum }_{q=1}^{nl}{G}_{q(ij)}[{V}_{i}^{2}+{V}_{j}^{2}-2{V}_{i}{V}_{j}\mathrm{ cos }({\delta }_{ij})]$$

where, ${G}_{q(ij)}$ is the conductance of branch *q* connected in between bus *i* and *j* and ${\delta }_{ij}={\delta }_{i}-{\delta }_{j}$, is the voltage angle difference.

#### Event 6: minimization of basic fuel cost with valve-point loading

**Figure 4:**
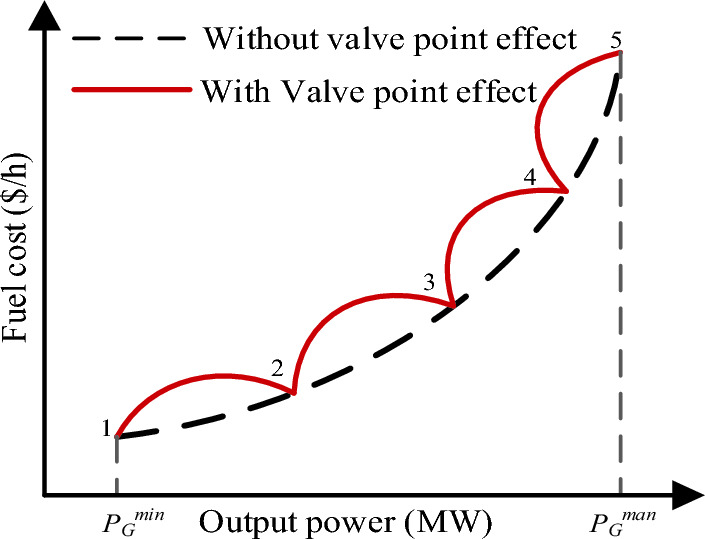
Single generators cost curve with and without valve point.

Valve-point loading wants to be measured for precise modeling and a more realistic cost of fuel vs generator output power (MW). Generation of power from multi-valve thermal engines shows variation in the fuel cost function, which is shown in the sinusoidal function. Such sinusoidal function is added to the fuel cost and resulting curve between output power (MW) vs fuel cost as shown in Fig. 4.

Mathematically, generator fuel cost considering valve-point loading is given by^9^:

24 $$f(x, n)={\sum }_{i=1}^{NG}{a}_{i}+{b}_{i}{P}_{{G}_{i}}+{c}_{i}{P}_{{G}_{i}}^{2}+|{d}_{i}\times \mathrm{sin }({e}_{i}\times ({P}_{{G}_{i}}^{\mathrm{ min }}-{P}_{{G}_{i}}))|$$

where the constants ${d}_{i}$ and ${e}_{i}$ are the valve point loading parameters, and their values are given in Table 2.

#### Event 7: simultaneous optimization of basic fuel cost and active power loss

The weighted sum approach is used to convert multi-objective optimization functions into single-objective optimization and is denoted as:

25 $$f(x, u)={\sum }_{i=1}^{NG}{a}_{i}+{b}_{i}{P}_{{G}_{i}}+{c}_{i}{P}_{{G}_{i}}^{2}+{\lambda }_{P}\times {P}_{loss}$$

whereas, active power loss ${P}_{loss}$ can be computed bsing Eq. (23) and the parameter ${\lambda }_{P}$ is equal to 40 as suggested in^8^.

#### Event 8: simultaneous optimization of voltage deviation and fuel cost

According to power quality, the voltage deviation index is the most important aspect, and it is minimized by enhancing the voltage profile. The cumulative voltage deviation (VD) function at the PQ nodes is described as:

26 $$VD=\left({\sum }_{p=1}^{NL}|{V}_{{L}_{p}}-1|\right)$$

The combined weighted sum of basic fuel cost and voltage deviation is given by:

27 $$f(x, u)=\left({\sum }_{i=1}^{NG}{a}_{i}+{b}_{i}{P}_{{G}_{i}}+{c}_{i}{P}_{{G}_{i}}^{2}\right)+{\lambda }_{VD}\times VD$$

where the weight factor ${\lambda }_{VD}$ is assigned a value of 100 as in^9^ and^8^.

#### Event 9: simultaneous optimization of voltage stability and fuel cost

Simultaneously, the minimization of basic fuel cost and maximization of voltage stability are converted into a single objective:

28 $$f(x, u)=\left({\sum }_{i=1}^{NG}{a}_{i}+{b}_{i}{P}_{{G}_{i}}+{c}_{i}{P}_{{G}_{i}}^{2}\right)+{\lambda }_{L}\times {L}_{\mathrm{ max}}$$

whereas, the parameter ${\lambda }_{L}$ is called a weight factor equal to 100 suggested by^8^ and *L*_*max*_ is computed by Eq. (21).

#### Event 10: simultaneous optimization of cost, emission, losses, and vd

In this event, simultaneously, four objectives are considered to minimize, and the combined fitness function is given:

29 $$f(x, u)=\left({\sum }_{i=1}^{NG}{a}_{i}+{b}_{i}{P}_{{G}_{i}}+{c}_{i}{P}_{{G}_{i}}^{2}\right)+{\lambda }_{E}\times Emission +{\lambda }_{VD}\times VD+{\lambda }_{P}\times {P}_{loss}$$

where, ${\lambda }_{E}=19,$ ${\lambda }_{VD}=21$ and ${\lambda }_{P}=22$ are the constant weights are considered the same as in^8^ to balance among the objective functions.

### IEEE 57-bus test system

To test the effectiveness of the C2oDE algorithm, the IEEE 57-bus system is considered. Four different events are considered to optimize with the C2oDE algorithm with two single objectives and the remaining two based on multi-objective, data given in Table 4.

**Table 4:** Data of IEEE 57-bus network under study.

Items	Quantity	Details
Buses (slack Bus)	57 (1)	^26^
Generator buses	7	1, 2, 3, 6, 8, 9, 12
Independent variable buses	50	–
Shunt VAR compensator buses	3	18, 25, 53
Total active and reactive demand	–	1250.8 MW, 336.4 MVAr
Branches	80	^26^
Tap changer transformer branches	17	19, 20, 31, 35, 36, 37, 41, 46, 54, 58, 59, 65, 66, 71, 73, 76, 80
Voltage range at slack and PV buses	7	[0.95–1.1] p.u
Voltage range at PQ buses	50	[0.94–1.06] p.u

#### Event 11: basic fuel cost minimization

In OPF, the basic objective is to minimize fuel cost, and mathematically, the function of fuel cost is the same as in Eq. (16). The coefficient of generator cost^26^ and emission^5^ are shown in Table 5.

**Table 5:** constant parameters of generator cost and emission of 57-bus network.

Generator	Bus	a	b	c	d	e	$\alpha$	$\beta$	$\gamma$	$\omega$	$\mu$
*G*_*1*_	1	0	20	0.0775795	18	0.037	4.091	− 5.554	6.49	0.0002	0.286
*G*_*2*_	2	0	40	0.01	16	0.038	2.543	− 6.047	5.638	0.0005	0.333
*G*_*3*_	3	0	20	0.25	13.5	0.041	6.131	− 5.555	5.151	0.00001	0.667
*G*_*4*_	6	0	40	0.01	18	0.037	3.491	− 5.754	6.39	0.0003	0.266
*G*_*5*_	8	0	20	0.0222222	14	4.258	− 5.094	4.586	0.000001	0.04	0.8
*G*_*6*_	9	0	40	0.01	15	0.039	2.754	− 5.847	5.238	0.0004	0.288
*G*_*7*_	12	0	20	0.0322581	12	0.045	5.326	− 3.555	3.38	0.002	0.2

#### Event 12: multi-objective optimization of fuel cost and vd

The weighted sum single objective optimization minimizes this event's basic fuel cost and VD. The fitness function in this study event is the same as in event 8 of IEEE 30-bus and mathematically is given by an Eq. (27).

#### Event 13: multi-objective optimization of voltage stability and fuel cost

The formulation of this event's weighted sum single objective function is the same as in event 9 of 30-bus. Also, lambda sub cap L is the same as in event 9.

#### Event 14: optimization of voltage deviation

In this event, the minimization of VD is considered the objective function of cumulative PQ buses and is calculated using Eq. (26).

### IEEE 118-bus system

Furthermore, a large-scale 118-bus standard IEEE test network is considered to test the superiority of the proposed C2oDE algorithm. A couple of single objective events are considered for this system. Table 6 gives the bus, branch, generator, and other related data of the 118-bus network.

**Table 6:** Data of IEEE 118-bus test system under study.

Items	Quantity	Details
Buses (slack Bus)	118 (69)	^26^
Generator buses	54	1, 4, 6, 8, 10, 12, 15, 18, 19, 24, 25, 26, 27, 31, 32, 34, 36, 40, 42, 46, 49, 54, 55, 56, 59, 61, 62, 65, 66, 69, 70, 72, 73, 74, 76, 77, 80, 85, 87, 89, 90, 91, 92, 99, 100, 103, 104, 105, 107, 110, 111, 112, 113, 116
Independent variable buses	130	–
Shunt VAR compensator buses	14	5, 34, 37, 44, 45, 46, 48, 74, 79, 82, 83, 105, 107, 110
Total active and reactive demand	–	4242 MW, 1439 MVAr
Branches	186	^26^
Tap changer transformer branches	9	8, 32, 36, 51, 93, 95, 102, 107, 127
Voltage range slack and PV buses		[0.95–1.1] p.u
Voltage range at PQ buses		[0.95–1.06] p.u

#### Event 15: basic fuel cost minimization

The constant parameters of fuel cost are taken from^26^, and the formulation of the fuel cost function is similar to event 1 of 30-bus.

#### Event 16: active power loss minimization

In this event, the minimization of real power loss is considered the objective function and calculated using Eq. (23).

## Proposed optimization algorithm

OPF is a constrained optimization problem, and how to solve constrained optimization problems has greater practical significance. Evolutionary algorithms (EAs) have involved noticeable attention in efficiently resolving practical constrained optimization problems in the past two decades. The constrained EAs have two main components: the search algorithm and the appropriate constrained handling method. Differential evolution (DE) is a popular EA; it has numerous attractive advantages to solving constrained optimization problems quickly because it is implemented, includes few control parameters, and achieves top rank in many computations^28^. Numerous DE variants have been applied in the literature to find solutions to constrained-type engineering problems. In this work, a constrained composite.

DE (C2oDE) global optimizer^25^ is proposed and added with two different CHTs to find the balance between constraints and objective functions. The framework of the proposed C2oDE optimization algorithm is introduced in the next section.

### C2oDE

In the C2oDE algorithm, differential vectors generate offspring^29^. Fundamentally, there are four stages in the proposed algorithm, in the first stage randomly generation of the initial population ${\overrightarrow{x}}_{i}^{t}(i\in \left\{1\dots NP\right\})$ in the range of lower and upper bound of search space. After that in the second stage, mutation operators are used for the generation of mutant vector ${\overrightarrow{v}}_{i}^{t}(i\in \left\{1\dots NP\right\})$, in this stage three type of mutation operators were used, such as.

1) current-to-rand/l

30 $${\overrightarrow{v}}_{i}^{t}={\overrightarrow{x}}_{i}^{t}+F\cdot ({\overrightarrow{x}}_{r1}^{t}-{\overrightarrow{x}}_{i}^{t})+F\cdot ({\overrightarrow{x}}_{r2}^{t}-{\overrightarrow{x}}_{r3}^{t})$$

2) Modified rand-to-best/l

31 $${\overrightarrow{v}}_{i}^{t}={\overrightarrow{x}}_{r1}^{t}+F\cdot ({\overrightarrow{x}}_{best}^{t}-{\overrightarrow{x}}_{r2}^{t})+F\cdot ({\overrightarrow{x}}_{r3}^{t}-{\overrightarrow{x}}_{r4}^{t})$$

**Figure 5:**
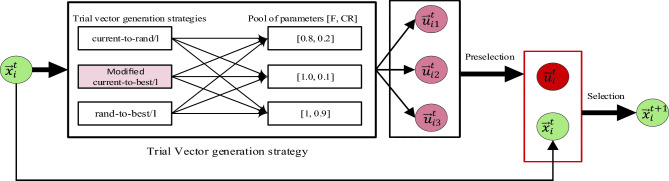
Framework of proposed C2oDE algorithm.

3) current-to-best/l

32 $${\overrightarrow{v}}_{i}^{t}={\overrightarrow{x}}_{i}^{t}+F\cdot ({\overrightarrow{x}}_{best}^{t}-{\overrightarrow{x}}_{i}^{t})+F\cdot ({\overrightarrow{x}}_{r1}^{t}-{\overrightarrow{x}}_{r2}^{t})$$

where, ${\overrightarrow{x}}_{r1}^{t}$ to ${\overrightarrow{x}}_{r5}^{t}$ are the mutually different decision vectors randomly selected from 1 to *NP* individuals, ${\overrightarrow{x}}_{best}^{t}$ The random differentiation shows the best solution for current generation t and rand. Each mutation vector has distinct features ; for example, the mutation vector given in Eq. (30) can explore the entire search space and increase diversity. However, Eqs. (31) and (32) accelerate the convergence to get information from the best individual. In the third step trial vector ${\overrightarrow{u}}_{i}^{t}$ is generated using a binomial crossover operator between each pair of ${\overrightarrow{v}}_{i}^{t}$ and ${\overrightarrow{x}}_{i}^{t}$ described as:

33 $$u_{{i,j}}^{t} = \left\{ {\begin{array}{*{20}l} {v_{{ij}}^{t} ,~} & {~if~rand_{j} < {\text{~}}CR{\text{~or~}}j = j_{{rand}} } \\ {x_{{ij}}^{t} ,~} & {otherwise} \\ \end{array} } \right.$$

where, ${x}_{i,j}^{t}, {u}_{i,j}^{t}$ and ${v}_{i,j}^{t}$ are the *j*^*th*^ dimension of ${\overrightarrow{x}}_{i}^{t}$, ${\overrightarrow{u}}_{i}^{t}$ and ${\overrightarrow{v}}_{i}^{t}$ Correspondingly, *CR* is the rate of crossover, and *J*_*rand*_ is the integer number randomly produce between 1 to *D*. Finally, in the fourth step the selection operator is applied among the ${x}_{i}^{t}$ and ${u}_{i}^{t}$ to find the candidate for the next population using Eq. (34) and Fig. 5 shows the framework of the proposed C2oDE algorithm.

34 $$\vec{x}_{i}^{{t + 1}} = \left\{ {\begin{array}{*{20}l} {\vec{u}_{i}^{t} ,~} & {iff\left( {\vec{u}_{i}^{t} } \right) < f\left( {\vec{x}_{i}^{t} } \right)} \\ {\vec{x}_{i}^{t} ,~} & {otherwise} \\ \end{array} } \right.$$

**Figure 6:**
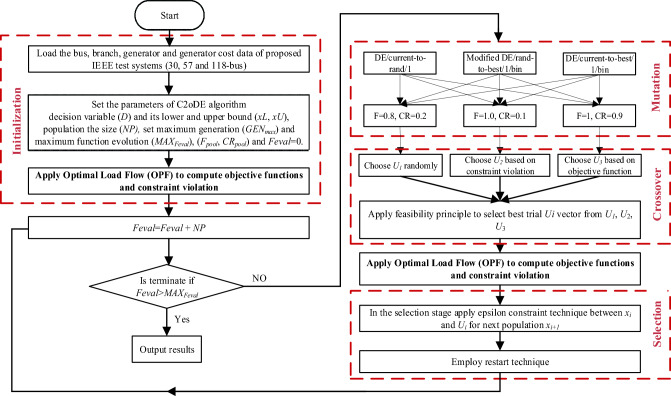
Flow chart for the implementation of C2oDE-FR-ECM.

It can be noticed from Fig. 5 that, for each target vector three off-springs are generated with distinct advantages of exploration and exploitation using trail vector generation strategy and pool of parameters. However, OPF problems are constrained optimization problems; therefore, there must be a compromise between objective function and constraint. Therefore, to balance constraint and objective function, two different CHTs are incorporated in this work at the phase of preselection and selection, as shown in Fig. 5. As stated in No Free Lunch (NFL)^20^, using various CHTs rather than single ones at different stages of EAs is better. Thus, the feasibility rule (FR) and ε constrained method (ECM) two CHTs are implemented with the proposed algorithm at the preselection phase and selection to select feasible trial vectors and populations for the next generation, respectively. OPF problems are very highly complicated. Therefore, a restart technique is used to avoid trapping into local optima, and it is triggered when the standard deviation of both the objective function or constraint violation is less than the assigned threshold value. The flow diagram of the proposed C2oDE-FR-ECM algorithm is given in Fig. 6. C2oDE maintains a population consisting of *NP* target vectors: ${\overrightarrow{x}}_{i}^{t}=\{{\overrightarrow{x}}_{1}^{t},{\overrightarrow{x}}_{2}^{t}, \dots ,{\overrightarrow{x}}_{NP}^{t} \}$, their objective function values:

${\varvec{f}}({\overrightarrow{{\varvec{x}}}}_{1}^{{\varvec{t}}}),{\varvec{f}}({\overrightarrow{{\varvec{x}}}}_{2}^{{\varvec{t}}}),$ $\dots$ , ${\varvec{f}}({\overrightarrow{{\varvec{x}}}}_{{\varvec{N}}{\varvec{P}}}^{{\varvec{t}}})$, and their degree of constraint violation: ${\varvec{G}}({\overrightarrow{{\varvec{x}}}}_{1}^{{\varvec{t}}}),$ ${\varvec{G}}({\overrightarrow{{\varvec{x}}}}_{2}^{{\varvec{t}}}),$ $\dots ,$ ${\varvec{G}}({\overrightarrow{{\varvec{x}}}}_{{\varvec{N}}{\varvec{P}}}^{{\varvec{t}}})$.

## Results and comparison

Various standard IEEE power system test networks were selected to judge the effectiveness of the proposed C2oDE algorithm. These include 30, 57, and 118-bus networks applying two different constraint handling techniques (CHTs) at various stages.

Table 7 summarizes the parameters of the proposed algorithm for the simulation of standard IEEE networks provided that values of F and CR are [0.8, 1.0, 1.0] and [0.2, 0.1, 0.9], respectively.

**Table 7:** PARAMETERS OF Proposed C2oDE ALGORITHM.

IEEE test system	Name of parameter (symbol)	Value(s)
30-bus	Population size (*NP*)	50
	Maximum iteration	100
	Maximum function evolution (${MAX}_{Feval}$)	15,000
57-bus	Population size (*NP*)	50
	Maximum iteration	200
	Maximum function evolution (${MAX}_{Feval}$)	30,000
118-bus	Population size (*NP*)	50
	Maximum iteration	1400
	Maximum function evolution (${MAX}_{Feval}$)	210,000

### Comparison among proposed chts

The C2oDE algorithm is compared and tested with the two most widely used CHTs, FR and ECM, at different places, such as at the preselection stage (to select the best trial vector) and selection (population for the next generation). Table 8 presents the statistical values over the 25 independent runs for the individual events of 1 to 14 using FR and ECM constraint handling methods. The columns of Table 8 show the best, mean, worst, and standard deviation of each event over 25 runs. Table 8 indicates that a single method cannot deliver the best statistical results in all the events. Therefore, this paper includes proposed CHTs in two stages to find a feasible trial vector and population for the next generation. Four different C2oDE variants were implemented considering two CHTs at different locations, i.e. C2oDE-FR, C2oDE-ECM, C2oDE-FR-ECM, and C2oDE-ECM-FR. Specifically, in C2oDE-FR and C2oDE-ECM, only the feasibility rule and ɛ constraint method were utilized for the best trail vector and population of the next iteration. However, in C2oDE-FR-ECM, the feasibility rule was used for finding the best trail vector, and ɛ constraint method was used to select the population for the next iteration while in C2oDE-ECM-FR, ECM for the trial vector, and FR was used to select candidates for the next iteration.

**Table 8:** Statistical summary of FR and ECM of event 1 to event 14.

Event no	C2oDE-FR				C2oDE-ECM				C2oDE-FR-ECM				C2oDE-ECM-FR			
	Best	Mean	Worst	Std dev	Best	Mean	Worst	Std dev	Best	Mean	Worst	Std dev	Best	Mean	Worst	Std dev
Event 1	800.4113	800.411	800.412	0.00029	800.4115	800.412	800.415	0.00067	800.4115	800.412	800.415	0.00096	800.4112	800.412	800.413	0.00032
Event 2	646.401	646.405	646.421	0.00510	646.403	646.410	646.445	0.00909	646.403	646.411	646.426	0.00574	646.402	646.407	646.450	0.00893
Event 3	0.13637	0.13634	0.13664	0.00006	0.13637	0.13649	0.13659	0.00006	0.13628	0.13646	0.13658	0.00005	0.13637	0.13646	0.13673	0.00006
Event 4	0.204817	0.20481	0.20481	0	0.204817	0.20481	0.20481	0	0.204817	0.20481	0.20481	0	0.204816	0.20481	0.20481	0
Event 5	3.08392	3.08403	3.08458	0.00012	3.08396	3.08415	3.08444	0.00011	3.08391	3.08405	3.08450	0.00013	3.0839	3.08411	3.08461	0.00019
Event 6	832.0700	832.072	832.087	0.00306	832.071	832.078	832.098	0.00620	832.071	832.074	832.087	0.00363	832.07	832.075	832.088	0.00481
Event 7	1040.111	1040.11	1040.12	0.00279	1040.112	1040.11	1040.122	0.00216	1040.11	1040.11	1040.12	0.00316	1040.11	1040.11	1040.12	0.00284
Event 8	813.110	813.119	813.200	0.01911	813.1116	813.1229	813.1860	0.013959	813.109	813.118	813.145	0.01026	813.110	813.117	813.131	0.00525
Event 9	814.1546	814.163	814.180	0.00609	814.155	814.1551	814.1551	814.1696	814.1543	814.162	814.179	0.00574	814.156	814.167	814.198	0.01015
Event 10	964.1173	964.118	964.123	0.00160	964.117	964.1204	964.125	0.001959	964.1172	964.118	964.122	0.00120	964.117	964.118	964.120	0.00084
Event 11	41,666.2	41,668.3	41,675.9	2.24494	41,666.4	41,667.47	41,672.11	1.316759	41,666.2	41,666.8	41,670.2	0.73187	41,666.2	41,667.44	41,680.18	2.56268
Event 12	41,774.4	41,775.2	41,778.5	0.75393	41,774.5	41,775.75	41,778.0	0.986349	41,774.6	41,775.5	41,777.9	0.94487	41,774.5	41,775.16	41,776.59	0.48211
Event 13	41,694.2	41,695.9	41,699.2	1.44858	41,694.3	41,695.49	41,701.64	1.579739	41,694.0	41,694.6	41,695.6	0.37272	41,694.1	41,695.47	41,701.65	1.53231
Event 14	0.58546	0.59163	0.59691	0.00278	0.58603	0.591117	0.601618	0.003876	0.58568	0.59043	0.60581	0.00393	0.58585	0.591937	0.599839	0.00370

The bold numbers shown in Table 8 are the best objective function values in a particular event obtained by methods. Furthermore, in Table 8, C2oDE-FR and C2oDE-FR-ECM outperform compared to C2oDE-ECM and C2oDE-ECM-FR. In contrast, C2oDE-ECM cannot beat any other variant in any study event, whereas C2oDE-ECM-FR only performs better in event1 and 4. On the other hand, FR and FR-ECM obtain the best fitness value, almost an equal number of events. Hence, selecting the proper CHTs for an OPF problem of various events is challenging because the objective function and constraints of OPF are non-linear. On the other hand, C2oDE-FR-ECM has the benefit of converging with the help of FR and exploring the entire search space to get better diversity with the help of ECM. Thus, the combination of FR and ECM at the different phases of the search algorithm, i.e., in C2oDE-FR-ECM, would attain the best value of the objective function or be close to the best fitness in most of the events. The subsequent subsections analyze and discuss the best results according to the objective functions of all the IEEE test systems.

### IEEE 30-bus test system

Table 9 shows the results of 30-bus system decision variables (i.e., state and control variables of event 1 to event 10). Column 2 and 3 of Table 9 displays the operating range of decision variables and in all the events, the results of these variables are within their allowable range and give the best value(s) of fitness considering one of the four proposed algorithms. In this work, the generator's output power in MW at the swing bus (*P*_*G1*_) and the MVAr rating of all the generators are considered the control variable and treated as inequality constraints during the optimization process. The allowable range of reactive power for all the generators is taken from MATPOWER^26^. Furthermore, simulation results obtained using the three variants of C2oDE by applying CHTs are presented in Table 10 (for single objective) and Table 11 (for multi-objective) compared with the recent methods of similar studies in the literature. Obtained results of proposed CHTs in which all the decision variables (dependent and independent) and constraints are within desirable limit however, in the approach of static penalty, some of these variables are violated and are highlighted with footnotes as shown in Table 10 and.

**Table 9:** Simulation results of event 1 to event 10 considering the best algorithm for a 30-bus network.

Parameter	Min	Max	Event 1	Event 2	Event 3	Event 4	Event 5	Event 6	Event 7	Event 8	Event 9	Event 10
Method			ECM-FR	FR	FR-ECM	ECM-FR	FR-ECM	FR>	FR	FR-ECM	FR-ECM	FR-ECM
*P*_G2_ (MW)	20	80	48.71265	54.99999	80	67.56307	79.9999	44.90895	55.60038	48.86460	48.73159	52.54368
*P*_G5_ (MW)	15	50	21.38571	24.15010	49.929	49.99999	50	18.48527	38.11460	21.62997	21.38919	31.46357
*P*_G8_ (MW)	10	35	21.22168	34.99988	34.972	34.99999	34.9999	10.00000	34.99998	22.29077	21.23591	34.99999
*P*_G11_ (MW)	10	30	11.90255	18.46280	29.964	29.99999	29.9999	10.00006	29.99999	12.22160	11.94318	26.75915
*P*_G13_ (MW)	12	40	12.00000	17.50430	12.007	39.99999	39.9999	12.00005	26.66520	12.00001	12.00052	20.96281
*V*_1_ (p.u)	0.95	1.10	1.083407	1.076121	1.0540	1.062643	1.06157	1.084165	1.068641	1.039886	1.082884	1.072411
*V*_2_			1.064326	1.061229	1.0509	1.056602	1.05736	1.061506	1.057917	1.024120	1.064111	1.058878
*V*_5_			1.033029	1.032719	1.0678	1.037187	1.03786	1.028460	1.034547	1.014304	1.033268	1.032103
*V*_8_			1.037638	1.041377	1.0569	1.043796	1.04414	1.035452	1.042653	1.005612	1.038894	1.040581
*V*_11_			1.089077	1.074367	1.0999	1.078060	1.07918	1.084049	1.083573	1.049336	1.098926	1.026010
*V*_13_			1.038980	1.041041	1.0782	1.050910	1.05257	1.052731	1.046200	0.987349	1.044608	1.010872
*Q*_c10_	0.0	5.0	0.661986	4.012780	3.5567	0.027334	0.02352	4.930163	0.318648	4.997871	0.132714	4.889569
*Q*_c12_			4.623068	4.653556	0.0460	3.156755	1.96798	1.631188	4.434062	0.000055	0.109537	4.849591
*Q*_c15_			4.114581	4.234578	0.0460	4.215265	4.34813	3.896941	4.099315	4.999996	4.056132	3.822877
*Q*_c17_			4.999902	4.998601	0.0527	4.999995	4.99991	4.999848	4.999927	4.30160	4.956622	4.999911
*Q*_c20_			3.928710	3.880312	0.0044	3.924083	3.86053	4.181339	3.826802	4.99997	3.650225	4.999284
*Q*_c21_			4.999993	4.999324	0.0447	4.999992	4.99995	4.999817	5	4.99999	4.999639	4.999999
*Q*_c23_			2.881190	2.872483	0.0128	2.996372	2.83785	3.099185	2.878838	4.99998	2.129788	4.309407
*Q*_c24_			4.999410	4.999246	0.0008	4.999984	4.99999	4.999993	4.999992	4.99999	4.997700	4.999998
*Q*_c29_			2.362086	2.307973	0.0006	2.280898	2.19882	2.472265	2.292495	2.63093	1.902285	2.605888
*T*_11_ (p.u)	0.90	1.10	1.070046	1.072383	1.0438	1.068853	1.06969	1.022621	1.055803	1.07091	1.035416	1.083315
*T*_12_			0.903210	0.902601	0.9000	0.900016	0.90000	0.980584	0.911555	0.90000	0.933892	0.959490
*T*_15_			0.964522	0.971061	1.0051	0.989224	0.98950	0.977150	0.981742	0.93788	0.963903	1.020102
*T*_36_			0.973223	0.973522	0.9639	0.975692	0.97528	0.977375	0.973868	0.97089	0.969430	1.004966
Fuel cost ($/h)	–	–	800.4112	646.40111	920.2534	944.3285	967.623	832.0708	859.0731	803.703152	800.41981	830.1861
Emission (t/h)	–	–	0.366392	0.283530	0.225365	0.204817	0.20726	0.437468	0.2288	0.363550	0.366159	0.253010
Ploss (MW)	–	–	9.005387	6.717099	4.50493	3.216795	3.08391	10.64437	4.525961	9.843499	9.002381	5.58624
V.D (p.u)	–	–	0.907200	0.921336	0.90040	0.900632	0.90460	0.863525	0.93148	0.0940676	0.940607	0.296498
L-index (max)	–	–	0.137988	0.137801	0.136283	0.138274	0.13816	0.139082	0.13778	0.148911	0.137344	0.147642
*P*_G1_ (MW)	50	200	177.1827	139.9999	81.03098	64.05372	51.4839	198.6500	102.545	176.2365	177.1019	122.2570
*Q*_G1_ (MVAr)	− 20	150	2.844612	− 0.48549	− 19.990	− 4.83944	− 5.0887	4.84590	− 3.2170	− 5.05825	1.940834	− 0.87367
*Q*_G2_ (MVAr)	− 20	60	20.24732	15.38954	− 19.986	7.564360	7.28340	15.4490	10.5911	14.95492	19.98522	12.75031
*Q*_G5_ (MVAr)	− 15	62.5	25.63524	24.98175	54.0075	21.66852	21.7331	24.1728	22.9242	46.65354	25.75591	23.28095
*Q*_G8_ (MVAr)	− 15	48.7	27.26701	27.98037	48.6904	27.69775	27.6035	28.0010	27.5810	38.55305	29.71277	27.39613
*Q*_G11_ (MVAr)	− 10	40	27.01666	21.05439	27.2552	22.99214	23.4502	19.8747	23.1381	25.03795	26.09648	13.55674
*Q*_G13_ (MVAr)	− 15	44.7	− 8.08520	− 6.46517	21.8333	1.698986	2.95095	2.1452	− 2.3857	− 14.99986	− 3.92963	− 0.45999

**Table 10:** Comparison of results of proposed algorithms with the past studies of the 30-bus single objective.

Event #	Method	Fuel cost ($/h)	Emission(ton/h)	*P*_*loss*_ (MW)	VD (p.u)	L-index
Event 1	FR	800.411384	0.36627	9.0021891	0.916732	0.1379627
	FR-ECM	800.411769	0.36647	9.0073043	0.923969	0.137890
	ECM-FR	800.411290	0.36639	9.0053876	0.907200	0.137988
	AGSO^4^	801.75	0.3703	–	–	–
	BSA^5^	799.0760^a^	0.3671	8.6543	1.9129^a^	0.1273
	SF-DE^6^	800.4131	0.36652	9.0104	0.92097	0.13786
	MSA^8^	800.5099	0.36645	9.0345	0.90357	0.13833
	ICBO^9^	799.0353^a^	–	8.6132	1.9652^a^	0.1261
	ARCBBO^12^	800.5159	0.3663	9.0255	0.8867	0.1385
	APFPA^13^	798.9144^a^	–	8.5800	1.9451^a^	–
	FHAS^14^	799.914^a^	–	–	1.5265^a^	–
	SKH^17^	800.5141	0.3662	9.0282	–	0.1382
	DE^7^	799.0827^a^	–	8.63	1.8505^a^	0.1277
Event 2	FR	646.40111	0.283530	6.71709	0.92133	0.13780
	FR-ECM	646.40372	0.283529	6.71765	0.93575	0.13768
	ECM-FR	646.40231	0.283537	6.71472	0.92743	0.13774
	BSA^5^	646.1504^a^	0.2833	6.6233	1.0273^a^	0.1378
	SP-DE^6^	646.4314	0.28351	6.7276	0.91253	0.13832
	MSA^8^	646.8364	0.28352	6.8001	0.84479	0.13867
	ICBO^9^	645.1668^a^	–	6.3828	1.8232^a^	0.1282
	GABC^11^	647.03	–	6.8160	0.8010	–
	LTLBO^15^	647.4315	0.2835	6.9347	0.8896	–
Event 3	FR	922.50411	0.2196	4.2548	0.92662	0.136346
	FR-ECM	920.25346	0.2253	4.5049	0.9004	0.136283
	ECM-FR	944.32857	0.2048	3.2167	0.90063	0.138274
	ECHT-DE^6^	917.5916	0.2252	4.5224	0.9110	0.13632
	SKH^17^	814.0100	0.3740	9.9056	–	0.1366
	DE^7^	915.2172^a^	–	3.626	2.1064^a^	0.1243
Event 4	FR	944.32776	0.204817	3.21682	0.899890	0.138313
	FR-ECM	944.33192	0.204817	3.21678	0.902163	0.138235
	ECM-FR	944.32857	0.204817	3.21679	0.900632	0.138274
	DSA^3^	944.4086	0.20583	3.2437	–	0.12734
	AGSO^4^	953.629	0.2059	–	–	–
	SF-DE^6^	944.3242	0.20482	3.2179	0.89617	0.13844
	MSA^8^	944.5003	0.20482	3.2358	0.87393	0.13888
	ARCBBO^12^	945.1597	0.2048	3.2624	0.8647	0.1387
Event 5	FR	967.6240	0.20726	3.08392	0.90314	0.13823
	FR-ECM	967.6239	0.20726	3.08391	0.90460	0.13816
	ECM-FR	967.6239	0.20726	3.08392	0.90499	0.13820
	DSA^3^	967.6493	0.20826	3.0945	–	0.12604
	SP-DE^6^	967.5962	0.20726	3.0844	0.90359	0.13832
	MSA^8^	967.6636	0.20727	3.1005	0.88868	0.13858
	ARCBBO^12^	967.6605	0.2073	3.1009	0.8913	0.1386
	APFPA^13^	965.6590^a^	–	2.8463a	2.0720^a^	–
Event 6	FR	832.0700	0.43746	10.6443	0.86352	0.13908
	FR-ECM	832.0708	0.43750	10.6468	0.84455	0.13912
	ECM-FR	832.0708	0.43754	10.6493	0.85802	0.13897
	BSA^5^	830.7779^a^	0.4377	10.2908	1.2050^a^	0.1363
	SF-DE^6^	832.0882	0.43730	10.6387	0.84935	0.13934
	ICBO^9^	830.4531^a^	–	10.2370	1.7450^a^	0.1289
	APFPA^13^	830.4065^a^	–	10.2178	1.8909^a^	–

^a^Voltage level at the PQ bus is violated.

**Table 11:** Comparison of results with the recent methods of proposed algorithms for 30-bus multi-objective.

Event #	Method	Fitness	Fuel Cost ($/h)	Emission (t/h)	Ploss (MW)	VD (p.u)	L-index (Max)
Event 7	FR	1040.111	859.0731	0.228881	4.52596	0.931488	0.13778
	FR-ECM	1040.112	859.0347	0.228902	4.52695	0.931709	0.13784
	ECM-FR	1040.113	859.0586	0.228907	4.52635	0.931267	0.13786
	ECHT-DE^6^	1040.151	858.867	0.22902	4.5321	0.93028	0.13796
	SF-DE^6^	1040.125	859.1458	0.2289	4.5245	0.92731	0.13785
	SP-DE^6^	1040.134	858.9319	0.22895	4.5301	0.92626	0.13781
	MSA^8^	1040.808	859.1915	0.22899	4.5404	0.92852	0.13814
	MFO^8^	1041.671	858.5812	0.22947	4.5772	0.89944	0.13806
Event 8	FR	813.1101	803.6978	0.3636726	9.84636	0.09412	0.148912
	FR-ECM	813.1099	803.70315	0.363550366	9.84349	0.09406	0.148911
	ECM-FR	813.1102	803.6922	0.3636732	9.84563	0.09418	0.148910
Event 8	BSA^5^	814.8994	803.4294	0.3546	9.3751	0.1147	0.14840
continue	ECHT-DE^6^	813.1742	803.7198	0.36384	9.8414	0.09454	0.14888
	SF-DE^6^	813.1956	803.4241	0.36424	9.7807	0.09772	0.14893
	SP-DE^6^	813.1959	803.4196	0.36324	9.7573	0.09776	0.14893
	MSA^8^	814.1545	803.3125	0.36344	9.7206	0.10842	0.14783
	MSA^8^	814.3541	803.7911	0.36355	9.8685	0.10563	0.14906
	ICBO^9^	813.5378	803.3978	–	9.7453	0.1014	0.14900
Event 9	FR	814.1545	800.41841	0.3664161	9.0086264	0.934922	0.13736
	FR-ECM	814.1542	800.41981	0.366159650	9.00238104	0.940607	0.13734
	ECM-FR	814.1564	800.41780	0.3663967	9.00883389	0.932287	0.13738
	ECHT-DE^6^	814.1708	800.4321	0.36585	9.0043	0.91244	0.13739
	SF-DE^6^	814.1649	800.4203	0.36592	8.9985	0.93846	0.13745
	SP-DE^6^	814.1841	800.4365	0.36517	8.9838	0.93706	0.13748
	MSA^8^	814.9378	801.2248	0.36106	8.9761	0.92655	0.13713
	FPA^8^	814.9067	801.1487	0.3718	9.3174	0.87563	0.13758
	ICBO^9^	811.8477^a^	799.3277^a^	–	8.6465	1.9961a	0.12520
	BSA^5^	812.9240^a^	800.3340^a^	0.3514	8.4904	1.9855a	0.12590
Event 10	FR	964.1172	830.1944	0.252987	5.585925	0.296461	0.147628
	FR-ECM	964.1171	830.1861	0.253010	5.586243	0.296498	0.147642
	ECM-FR	964.1171	830.2012	0.2529848	5.5858021	0.296265	0.147628
	ECHT-DE^6^	964.1331	830.1156	0.25293	5.5894	0.29738	0.14748
	SF-DE^6^	964.1254	830.1366	0.25313	5.5887	0.29653	0.14756
	SP-DE^6^	964.1234	830.2123	0.25294	5.5857	0.29615	0.14756
	MSA^8^	965.2905	830.639	0.25258	5.6219	0.29385	0.14802
	MFO^8^	965.8077	830.9135	0.25231	5.5971	0.33164	0.14556

Table 11. During the optimization process, voltages at the PQ, buses are often found critical, such as near the upper limit (0.95–1.05 p.u). Frequently, failure of the power system components appears due to overvoltage, and it is highly undesirable.

On the other hand, the Voltage deviation (VD) of the IEEE 30-bus system would be 1.2 p.u (24 × 0.05) if the value of voltage level at all load buses is under the permissible limit. However, in the literature in many cases, VD is more than a permissible specified value and is also highlighted with footnotes, as shown in Table 10 and.

**Figure 7:**
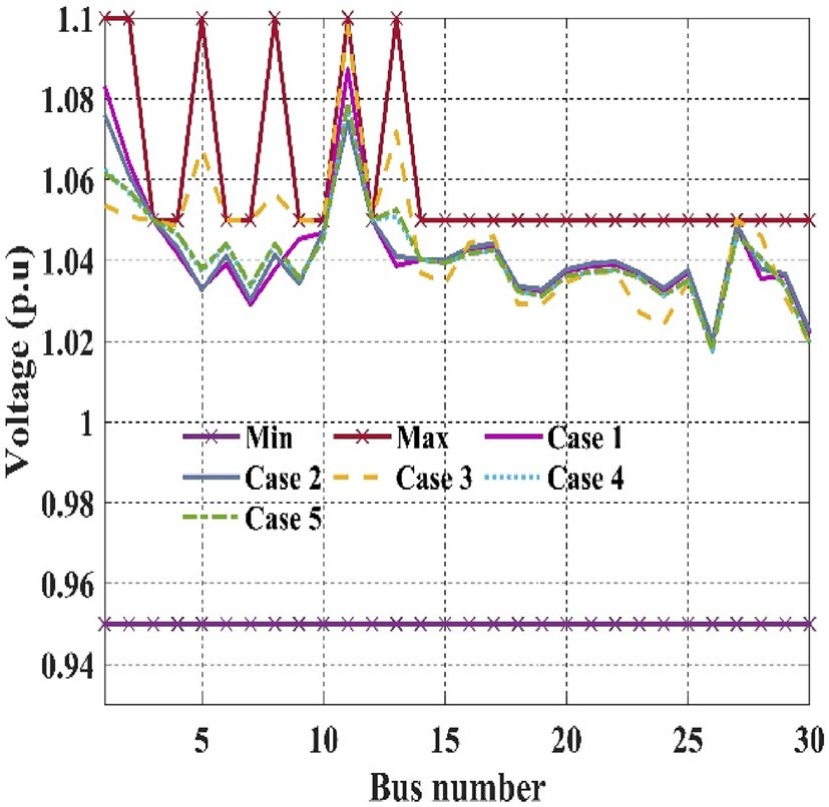
Event-1 to event-5: Voltage profile of best solution For IEEE 30-bus systems.

**Figure 8:**
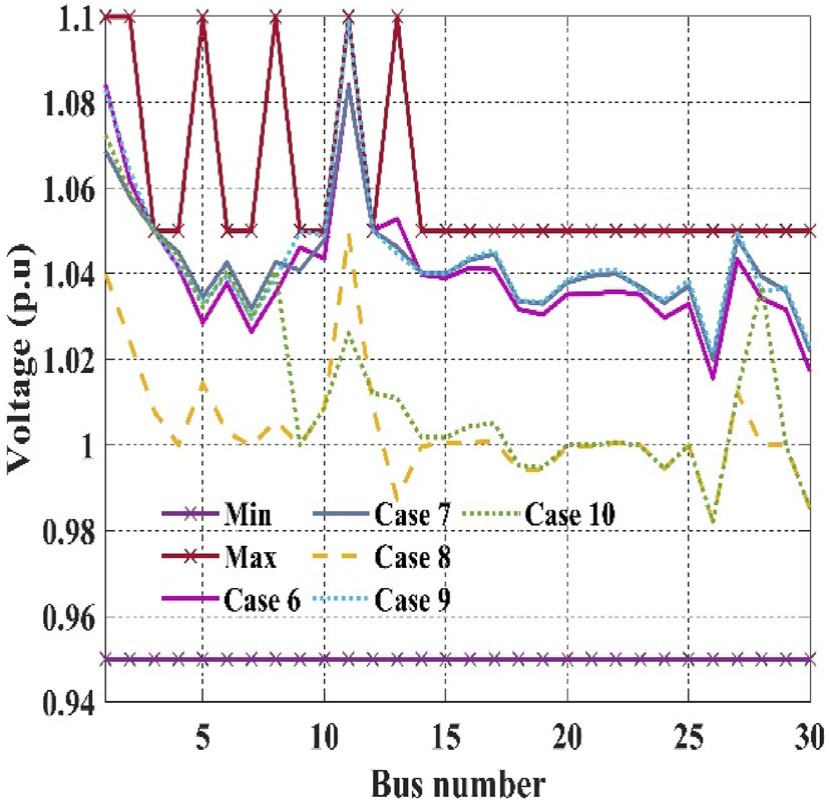
Event-6 to event-10: Voltage profile of best solution For IEEE 30-bus systems.

Table 11. Furthermore, the main goal of this work is to prove effectiveness by merely considering statistical results and establishing the strict agreement of system constraints using various CHTs. It is noticed from Table 10 that values of the objective function in event 1 using FR and ECM-FR give 800.411290$/h and 800.411384, respectively, satisfying all the inequality constraints. In event 2 C2oDE-FR finds the minimum cost of 646.40111 $/h among various CHTs considering the multi-fuel effect however, in event 3 in which fitness function is considered to minimize the maximum L-index (*L*_*max*_) of PQ buses, FR-ECM obtained the best simulation result of 0.13628 in comparison to the algorithms of past studies. In event 4, the minimization of emissions in (t/h) is 0.204817, almost the same in all the proposed CHTs. Also, the algorithms reported in the literature include SF-DE^6^, MSA^8^, and ARCBBO^12^ whereas, in event 5 minimization of active power losses, FR-ECM and FR give the best results of 3.08391 MW and 3.08392 MW compared with the other techniques shown in Table 10. Voltage waveforms of the 30-bus network are given in Figs. 7 and 8 and it shows that the output value of voltage (p.u) is within the range of minimum and maximum value without violating any of the constraints.

The fitness function of fuel cost minimization considering valve-point loading proposed in event 6, in which C2oDE-FR obtained the best result of 832.0700 $/h is high compared to basic fuel cost in event 1. However, in events, 7–10 weighted sum multi-objective optimization of various functions is proposed in which the combined effect of various single objective functions decides the output results of optimization algorithms. For example, in event 7, a higher weight charge of fuel cost was preferred to minimize more fuel cost than power loss.

**Figure 9:**
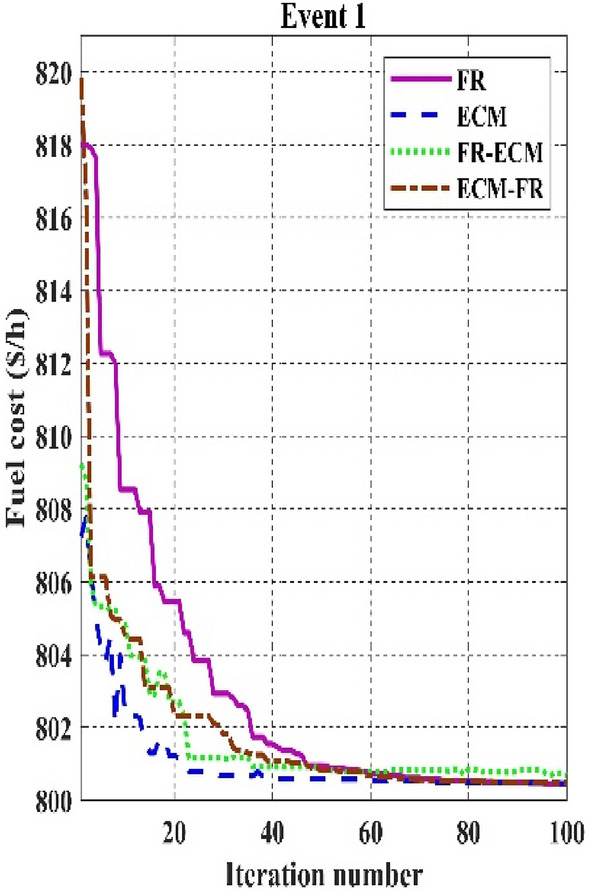
Convergence curves of comparative CHTs of event-1.

**Figure 10:**
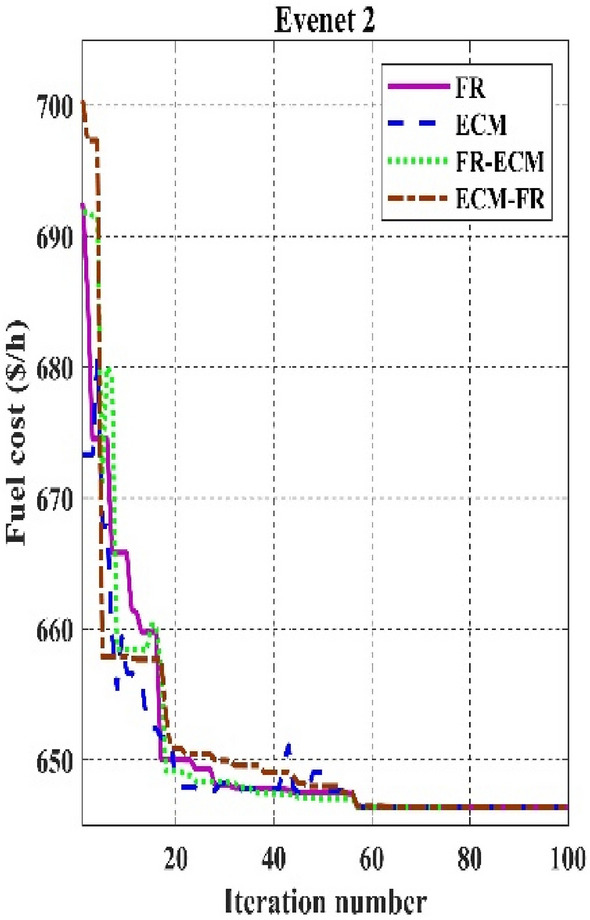
Convergence curves of comparative CHTs of event-2.

**Figure 11:**
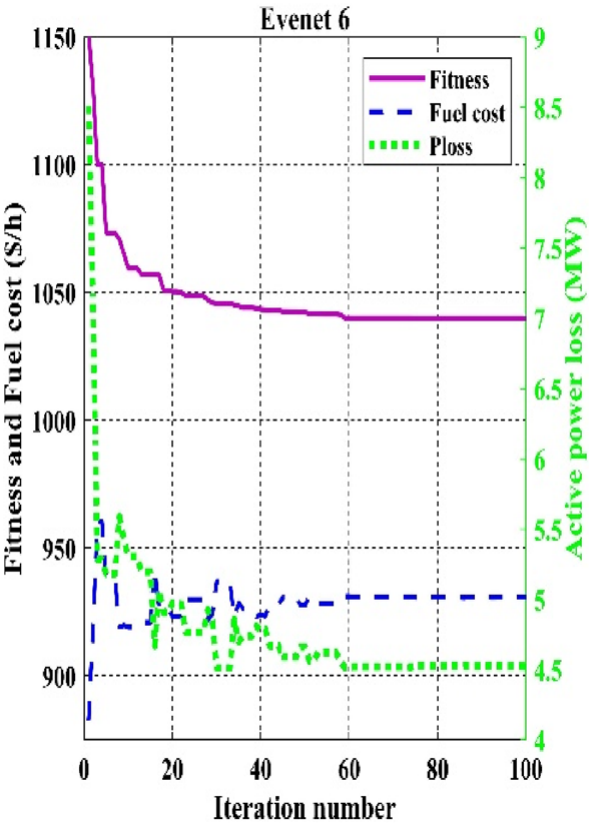
Convergence curves of comparative CHTs of event-6.

Table 11 shows that the single algorithm FR, FR-ECM, or ECM-FR is not able to find the best value of fitness in all the events. In event 7, C2oDE-FR gives the global minimum of combined fitness of 1040.11188 compared to other methods. Furthermore, in events 8 to event 10, obtained values of combined multi-objective functions are minimal in FR-ECM compared to all the algorithms. Furthermore, the convergence curve of C2oDE using two CHTs at different phases for events 1, 2, and 6 considering fuel cost as the objective function are indicated in Figs. 9, 10, 11, respectively.

**Figure 12:**
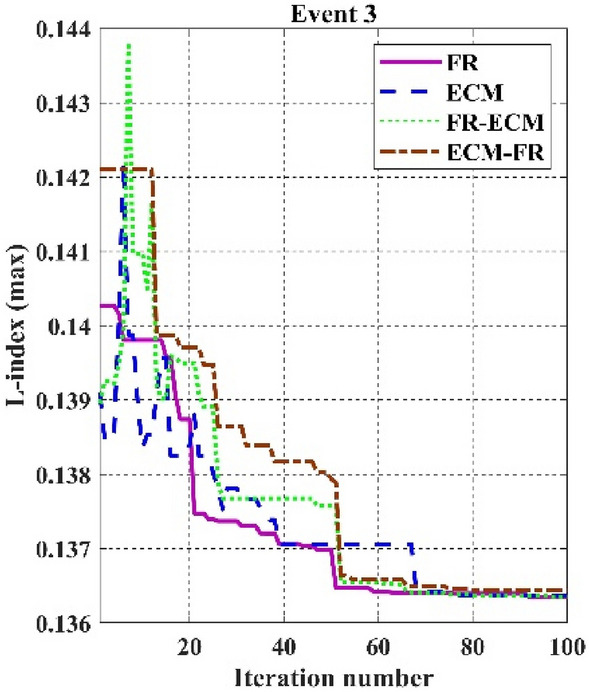
Convergence curves of comparative CHTs of event-3 for 30-bus.

**Figure 13:**
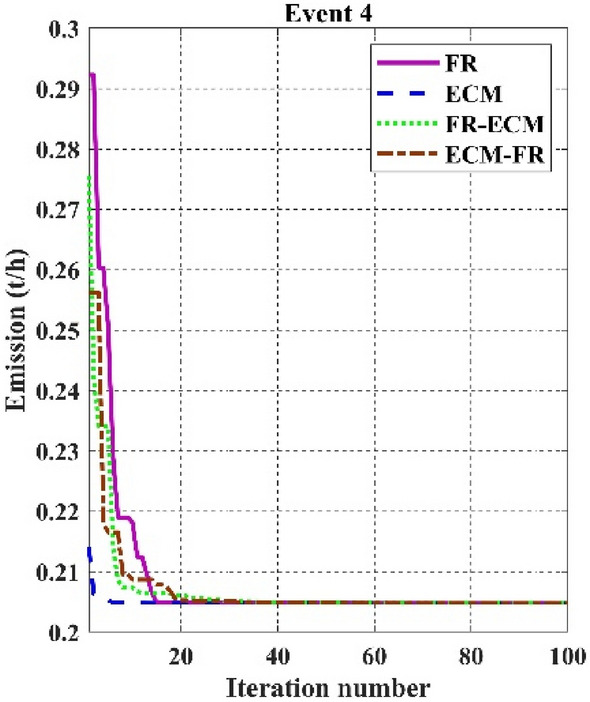
Convergence curves of comparative CHTs of event-4 for 30-bus.

**Figure 14:**
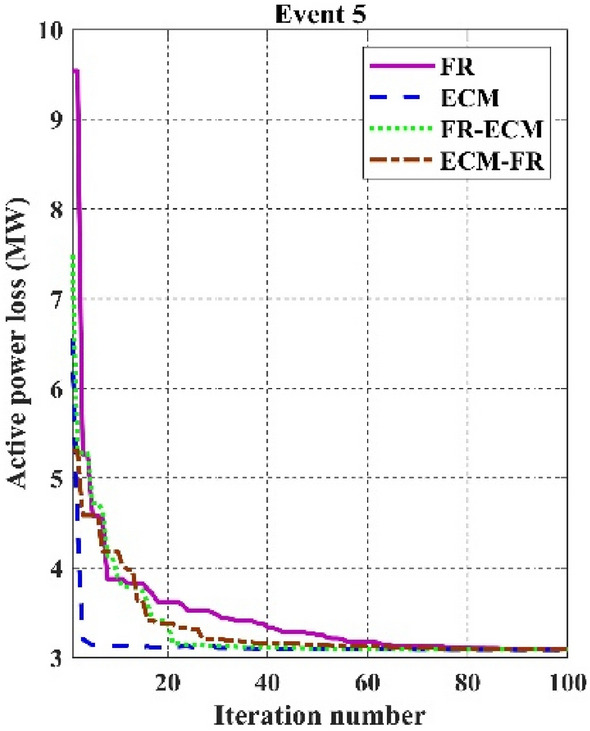
Convergence curves of comparative CHTs of event-5 for 30-bus.

**Figure 15:**
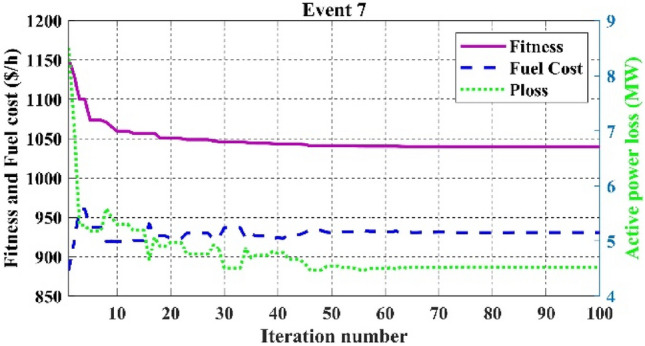
Convergence curves of event-7 (C2oDE-FR) for 30-bus.

**Figure 16:**
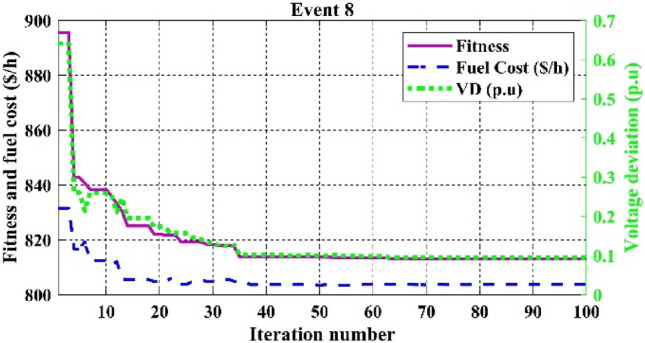
Convergence curves of event-8 (C2oDE-FR-ECM) for 30-bus.

**Figure 17:**
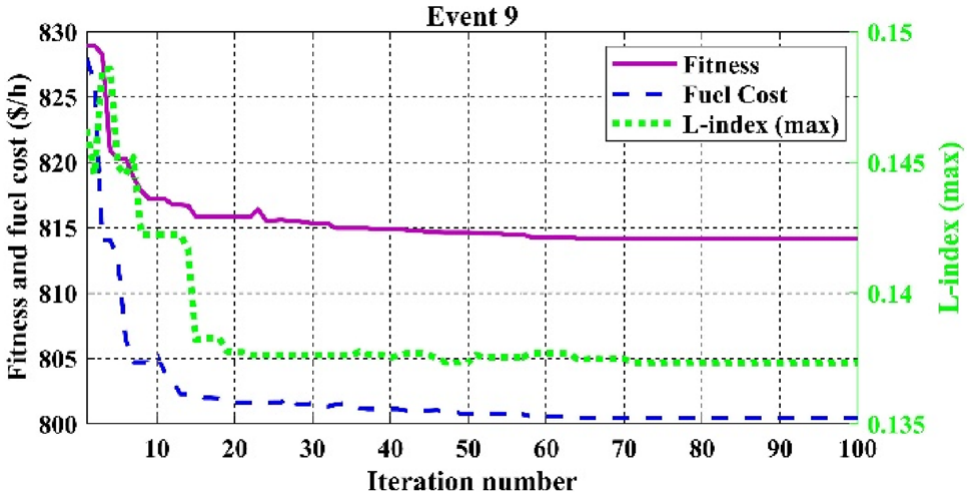
Convergence curves of event-9 (C2oDE-FR-ECM) for 30-bus.

**Figure 18:**
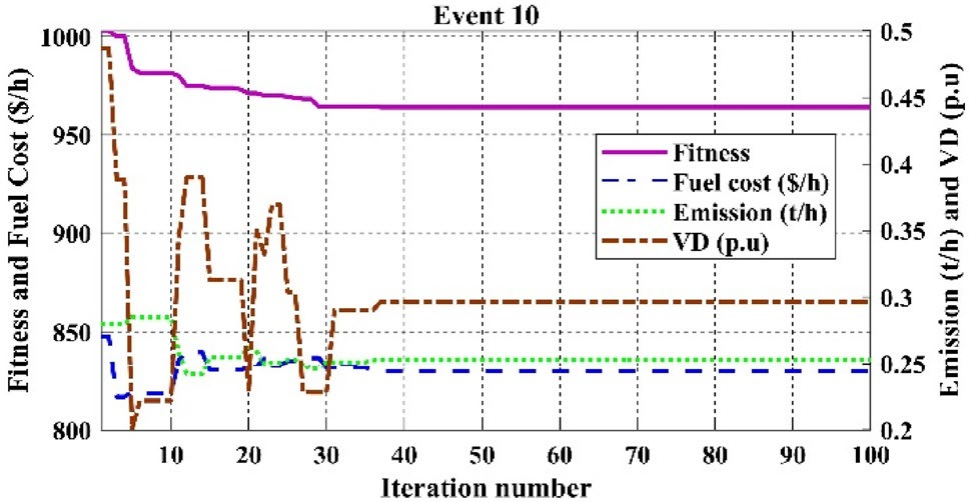
Convergence curves of event-10 (C2oDE-FR-ECM) for 30-bus.

Among the different CHTs, the convergence speed is not strangely different, though rapid and smooth convergence is observed in both FR and FR-ECM. Figures 12, 13, 14 give the convergence curve of event 3 to event 5, respectively. In event 3, the voltage stability index indicator is scrutinized in the fitness function in which the convergence curve is uneven because of the nature of the objective function. Moreover, Figs. 15, 16, 17, 18 show the convergence curve of multi-objective optimization events. The convergence curve of only the best fitness value of CHTs is shown in Figs. 15, 16, 17, 18 for clear visibility and the irregularities between objective functions during convergence due to non-linear relationships among the fitness and independent variables.

### IEEE 57-bus test system

The solution of decision variables (i-e. dependent and control) of the 57-bus network and the simulation results of best objective functions among all the methods are demonstrated in Table 12.

**Table 12:** Simulation results of the best algorithm for 57-bus network.

Parameters	Min	Max	Event 11	Event 12	Event 13	Event 14	Parameters	Min	Max	Event 11	Event 12	Event 13	Event 14
Method			FR-ECM	FR	FR-ECM	FR	T_46_ (p.u.)	0.90	1.10	0.95938	0.93799	0.95788	0.91940
PG_2_ (MW)	30	100	90.1565	88.1932	90.0784	93.3671	T_54_			0.91393	0.90005	0.91009	0.90000
PG_3_	40	140	45.0229	45.0262	44.9526	84.1611	T_58_			0.98115	0.96771	0.97925	0.92858
PG_6_	30	100	71.3654	71.4852	70.7965	30.0439	T_59_			0.96506	0.96646	0.96371	0.98846
PG_8_	100	550	460.683	460.421	461.273	274.863	T_65_			0.97649	0.98384	0.97394	1.02150
PG_9_	30	100	95.2681	97.7248	95.5262	99.9675	T_66_			0.93781	0.93612	0.93612	0.90000
PG_12_	100	410	360.177	360.758	360.320	365.628	T_71_			0.97438	0.97024	0.97220	0.96681
V_1_ (p.u.)	0.95	1.10	1.0665	1.03347	1.06459	1.00444	T_73_			0.99484	0.99670	0.99783	1.00903
V_2_			1.06390	1.03172	1.06215	1.00609	T_76_			0.96010	0.94092	0.96557	0.90000
V_3_			1.05518	1.02677	1.05412	1.01118	T_80_			1.00457	1.01062	0.99962	0.99618
V_6_			1.05973	1.04270	1.05960	1.00360	Fuel cost ($/h)			41,666.2	41,697.5	41,666.2	46,007.0
V_8_			1.07540	1.06284	1.07539	1.02754	Emission (t/hr)			1.3543	1.35461	1.35640	1.28646
V_9_			1.05044	1.02831	1.04901	1.01456	P_loss_ (MW)			14.8698	15.5854	14.8805	21.1843
V_12_			1.05210	1.01791	1.04898	1.04116	VD (p.u.)			1.71752	0.76847	1.70200	0.58546
Q_c18_ (MVAr)	0	20	7.58169	6.69655	7.69420	0.00351	L-index (max)			0.27862	0.29317	0.27869	0.30140
Q_c25_			13.5669	15.6555	13.7011	19.9998	P_G1_ (MW)	0	576	142.995	142.775	142.732	323.952
Q_c53_			12.4177	16.2810	12.2559	19.9982	Q_G1_ (MVAr)	− 140	200	46.8982	42.6812	46.8863	− 48.657
T_19_ (p.u.)	0.90	1.10	0.94060	0.99181	1.02575	0.92397	Q_G2_	− 140	200	49.9795	49.9999	49.9258	49.9918
T_20_			1.02197	0.99278	0.95456	1.03120	Q_G3_	− 10	60	31.0003	32.0709	32.2192	59.9906
T_31_			1.01187	0.99231	1.00920	0.97055	Q_G6_	− 8	25	− 7.1320	− 4.4612	− 6.5401	− 7.9924
T_35_			1.02515	1.02299	0.94884	1.06276	Q_G8_	− 140	200	50.9866	73.1770	53.9570	53.9784
T_36_			1.00895	0.99187	1.09712	1.07872	Q_G9_	− 140	200	8.99564	8.99999	8.98084	8.99543
T_37_			1.03387	1.02340	1.03227	1.00742	Q_12_	− 150	155	58.6397	42.9339	55.0253	154.920
T_41_			0.99535	1.01861	0.99540	0.99791							

Table 12 clearly shows that the decision variables are within the desirable range. However, Table 13 compares all CHTs (FR, FR-ECM, ECM-FR) with the recent literature methods. Minimum and maximum values of a few generators' MVAr ratings are relatively narrow and taken from^26^ even though proposed CHTs dully satisfied the generator reactive power limit. Further, the IEEE 57-bus system consists of 50 PQ buses, and the voltage level of feasible solutions of these buses must be within [0.94 to 1.06] p.u range and the cumulative VD would be (50 × 0.06) 3 p.u. The values of VD found to be more than 3 p.u are marked with a footnote in one reference in which static penalty function is used as CHTs. In events 12 and 14 among four CHTs, the results of C2oDE-FR are best; on the other hand, in events 11 and 13 C2oDE-FR-ECM outperformed among all the proposed CHTs, providing all the constraints are within feasible search space. In most of the events, according to the minimization of objective functions of IEEE 57-bus systems, C2oDE-FR and C2oDE-FR-ECM outperform in comparison to the methods of past studies. In event 11 the best value of the objective function is 41,666.2413 $/h, the lowest values comparison to the methods as shown in Table 13 also the power loss (14.86981151 MW) is best compared to the method available in the literature. Event 12 is the multi-objective, considering fuel cost and VD by C2oDE-FR is 41,774.422, which is close to the value given by SP-DE^6^.

**Table 13:** Comparison of proposed algorithms with the past studies of IEEE 57-bus system.

Event #	Algorithm	Fitness	Fuel Cost ($/h)	Emission (t/h)	Ploss (MW)	VD (p.u)	L-index (p.u)
Event 11	FR	41,666.2483	41,666.2483	1.35477	14.8576	1.70050	0.279264
	FR-ECM	41,666.2413	41,666.2413	1.35436	14.8698	1.71752	0.278628
	ECM-FR	41,666.2012	41,666.2012	1.35219	14.8386	1.68709	0.279045
	ECHT-DE^6^	41,670.56	41,670.56	1.36230	14.9479	1.50319	0.28886
	SF-DE^6^	41,667.85	41,667.85	1.35816	14.8864	1.64209	0.27971
	SP-DE^6^	41,667.82	41,667.82	1.350	14.9090	1.54367	0.28123
	MSA^8^	41,673.72	41,673.72	1.9526	15.0526	1.5508	0.28392
	ICBO^9^	41,697.33	41,697.33	–	15.5470	1.3173	0.27760
	DSA^3^	41,686.82	41,686.82	–	–	1.0833	0.24353
	ARCBBO^12^	41,686	41,686	–	15.3769	–	–
	APFPA^13^	41,628.75^a^	41,628.75^a^	–	14.0470	3.5571^a^	–
	LTLBO^15^	41,679.55	41,679.55	–	15.1589	–	–
	MICA-TLA^19^	41,675.05	41,675.05	–	15.0149	1.6161	–
	DE^7^	41,682	41,682	–	–	–	–
Event 12	FR	41,774.422	41,697.57549	1.354616072	15.58540	0.768472	0.29317
	FR-ECM	41,774.615	41,695.78343	1.354867694	15.57094	0.788316	0.29261
	ECM-FR	41,774.495	41,697.47045	1.352955505	15.58260	0.770246	0.29254
	ECHT-DE^6^	41,776.48	41,694.82	1.3597	15.5806	0.81659	0.29198
	SF-DE^6^	41,775.09	41,697.52	1.35769	15.5616	0.77572	0.29262
	SP-DE^6^	41,774.75	41,697.50	1.3550	15.5897	0.77253	0.29228
	MSA^8^	41,782.80	41,714.98	1.9551	15.9214	0.6782	0.29533
	DSA^3^	41,775.60	41,699.40	–	–	0.7620	0.2471
	MFO^8^	41,786.66	41,718.87	2.0149	16.2189	0.6780	0.29525
	MICA-TLA^19^	42,013.08	41,959.18	–	19.909	0.5390	–
Event 13	FR	41,694.219	41,666.361	1.3545	14.8485	1.726307	0.27858
	FR-ECM	41,694.089	41,666.220	1.3564	14.8805	1.702002	0.27869
	ECM-FR	41,694.180	41,666.340	1.3551	14.8718	1.734348	0.27840
	ECHT-DE^6^	41,699.25	41,671.09	1.3609	15.0275	1.56188	0.28152
	SF-DE^6^	41,695.55	41,667.53	1.3576	14.8963	1.61174	0.28022
	SP-DE^6^	41,696.54	41,668.45	1.35524	15.012	1.60803	0.28092
	MSA^8^	41,703.48	41,675.99	1.9188	15.0026	1.7236	0.27481
	DSA^3^	41,785.05	41,761.22	–	–	1.0573	0.2383
	MFO^8^	41,707.66	41,680.19	1.9192	15.1026	1.7245	0.27467
Event 14	**FR**	0.5854631	46,007.051	1.28646	21.1843	0.58546	0.30140
	FR-ECM	0.5856809	43,805.627	1.15070	15.7835	0.58568	0.30146
	ECM-FR	0.5858559	47,905.304	1.33543	20.9817	0.58585	0.30150
	ECHT-DE^6^	0.60416	46,813.22	1.3379	19.0821	0.60416	0.3008
	SF-DE^6^	0.59584	45,246.02	1.23453	18.4697	0.59584	0.30135
	SP-DE^6^	0.59267	45,549.49	1.2898	18.4275	0.59267	0.30052
	APFPA^13^	0.8909	43,485.93	–	12.1513	0.8909	–
	KHA^16^	0.5810^b^	42,006.44	–	–	0.5810^b^	0.2985
	DE^7^	0.5839^b^	–	–	–	0.5839^b^	–

^a^Voltage on the PQ bus is violated, the solution is infeasible.

^b^Considered the large limits of shunt VAR compensators.

**Figure 19:**
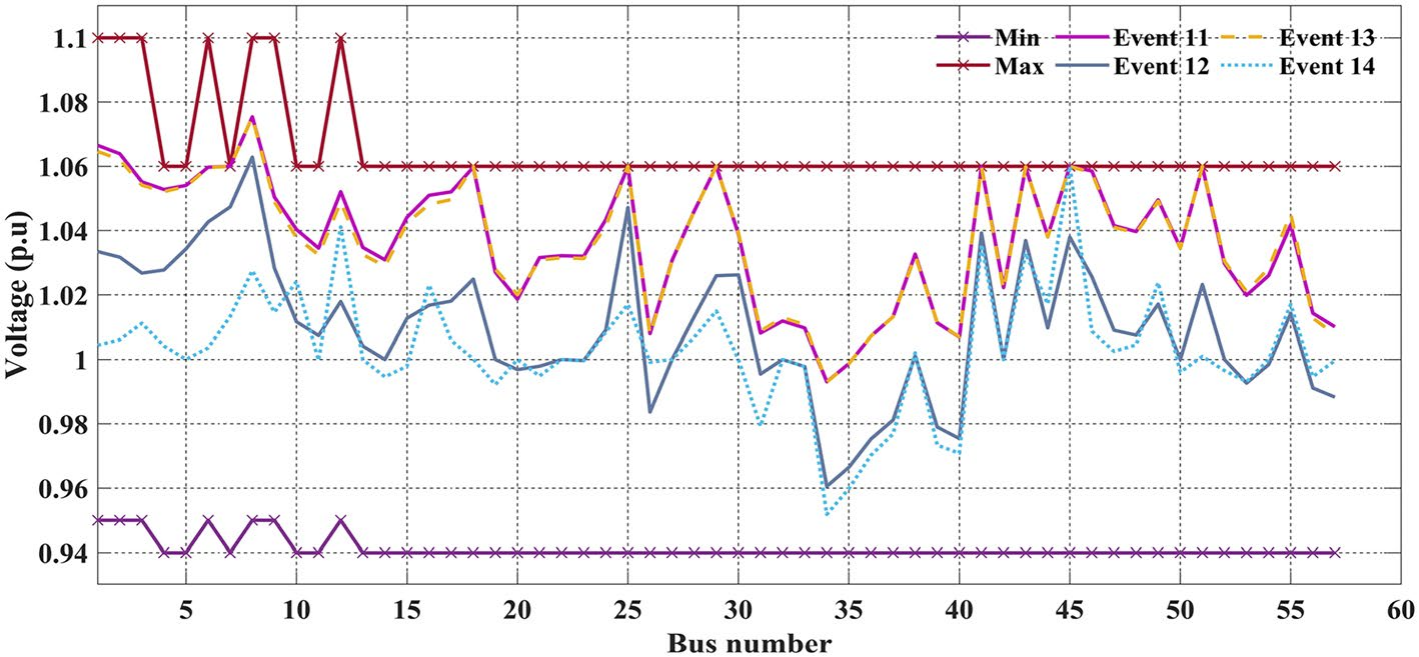
Event-11 to event-14: Voltage profile of best solution For IEEE 57-bus systems.

Further, multi-objective voltage stability and fuel cost are considered in event 13, in which fuel cost (41,694.089) seems better compared to event 12. In event 14 C20DE-FR outperformed APFPA^13^, however with the expense of fuel cost compared to the previous study. Larger values of shunt VAR compensators (30 MVAr) have been seen in^16^ and^7^, hence comparison with the present study is not valid. Figure 19 shows the voltage profiles of the best solution among the different CHTs of event 11 to event 14 for the 57-bus system.

**Figure 20:**
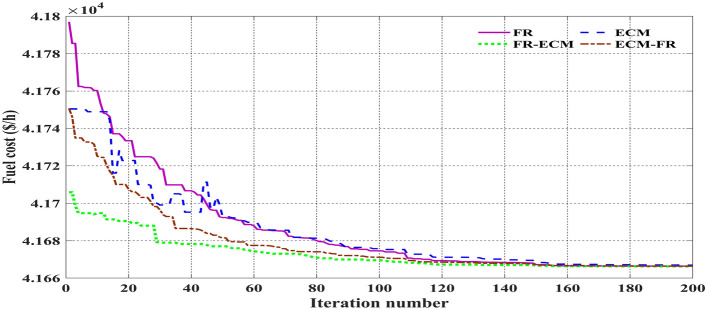
Relative convergence curves of event-11 (C2oDE-FR-ECM) for IEEE 57-bus.

**Figure 21:**
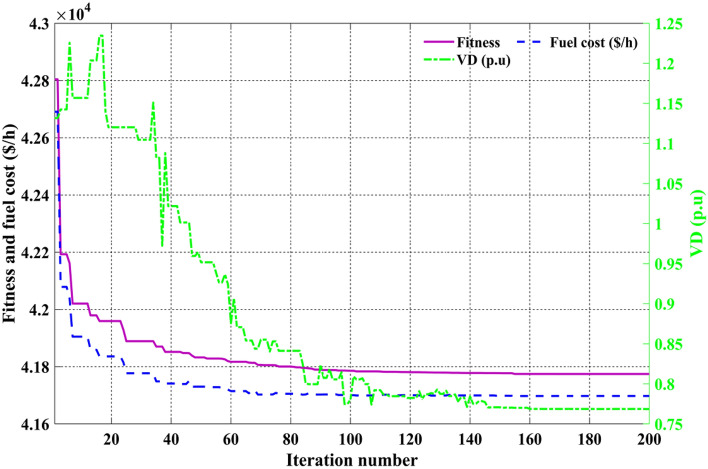
Convergence curves of event-12 (C2oDE-FR) for 57-bus.

**Figure 22:**
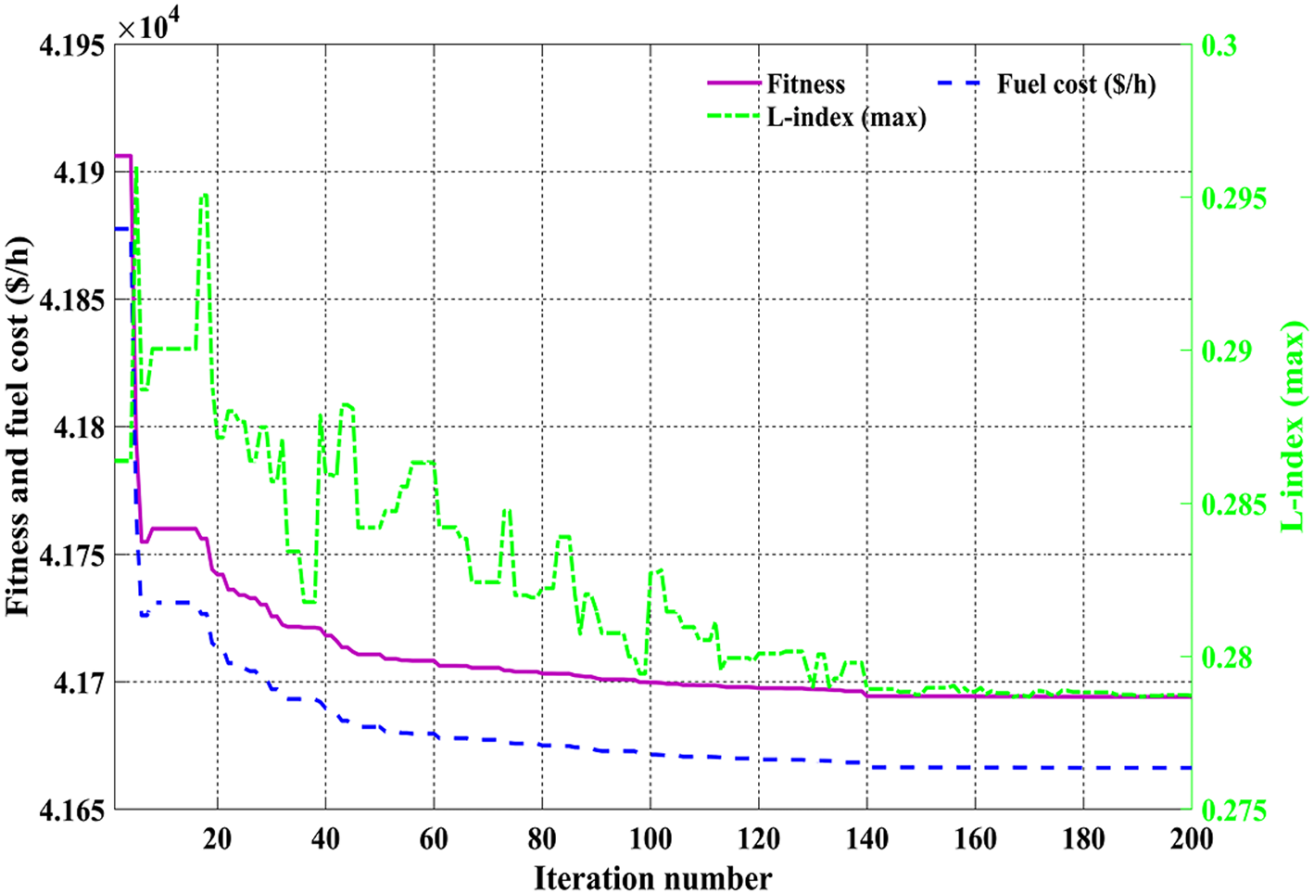
Convergence curves of event-13 (C2oDE-FR-ECM) for 57-bus.

Figure 19 clearly shows that the operating value of the voltage at all the buses is within the minimum and maximum range, such as satisfying voltage constraints so that no bus experiences overvoltage, whereas, in some buses, the voltage level is close to the upper bound. Figure 20 shows the convergence curves of applied CHTs. As compared to other methods, C2oDE-FR-ECM converges faster in event 11 and attains a feasible solution; subsequently, a considerable number of objective function evaluations due to generators' reactive power limits and in the optimization process, convergence of the actual solution starts when the optimization algorithm attains the feasible search space. Further, the clear convergence diagram of the multi-objective optimization fitness function of event 12 and event 13, in which only the best methods are presented, is shown in Figs. 21 and 22, respectively.

**Figure 23:**
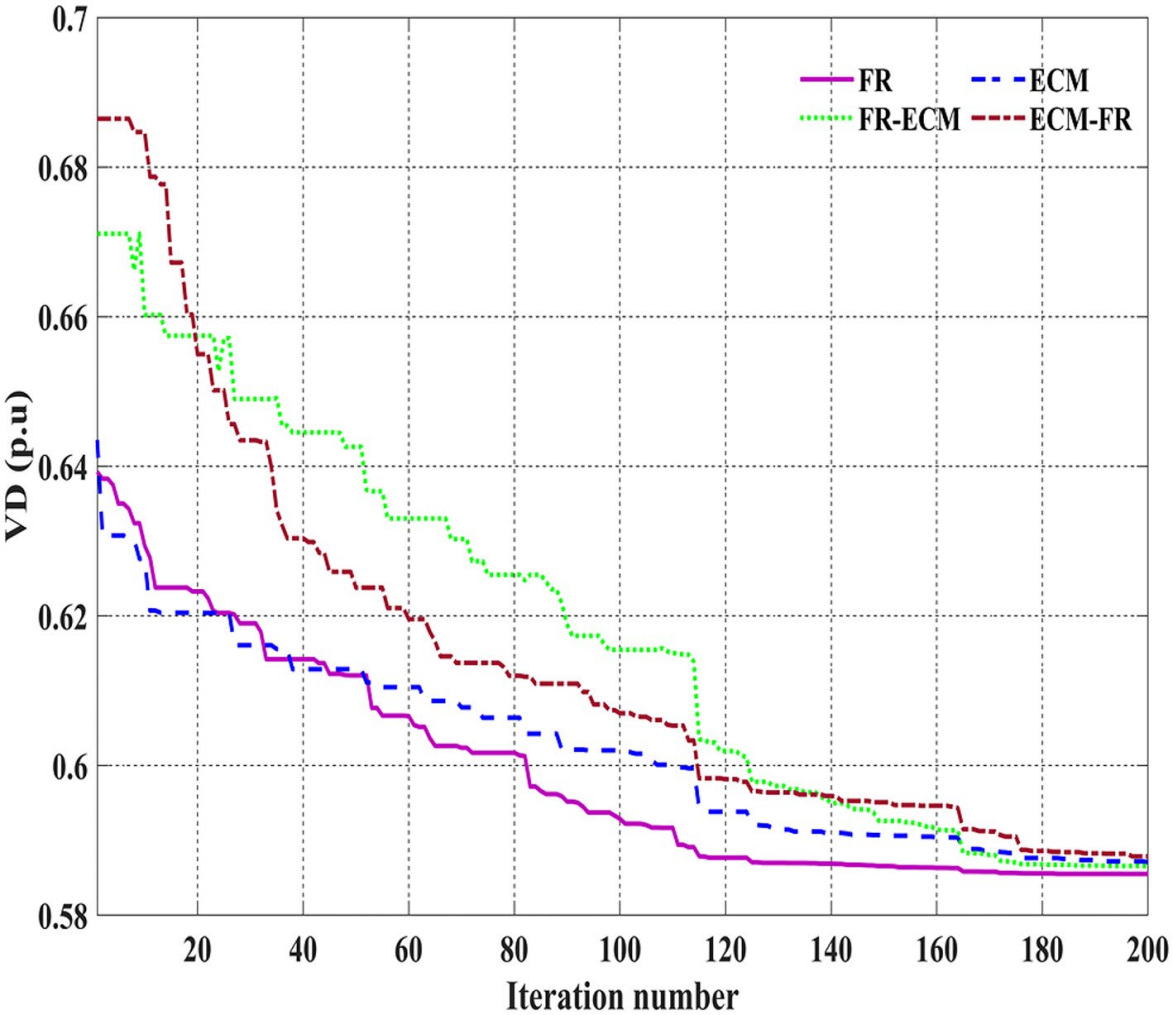
Convergence curves of event-14 (C2oDE-FR) for IEEE 57-bus.

We can notice from the above figures that the convergence curves of voltage deviation and L-index are non-smooth in multi-objective events. Figure 23 shows the convergence curve of single objective optimization (voltage deviation) in which all methods need many fitness function evaluations to seek the global optimum solution because of the non-linear relation between bus voltage and independent variables in the 57-bus tests network.

### IEEE 118-bus test system

Generally, for an increased number of variables, the performance of C2oDE-FR-ECM is found to be superior. Hence, in the large-scale 118-bus test network, an effective combined FR-ECM constraint technique is proposed to show the superiority and scalability of the proposed algorithm. Furthermore, the minimization of basic fuel cost (Event 15) and real power losses (Event 16) is considered the objective functions of this system. Table 14 shows the calculated parameters and control variables of the best solution found using C2oDE-FR-ECM.

**Table 14:** Simulation results of C2oDE-FR-ECM algorithm for IEEE 118-bus.

Control Variables	Min–Max	Event 15	Event 16	Control Variables	Min–Max	Event 15	Event 16	Control Variables	Min–Max	Event 15	Event 16
*PG*_*1*_ (MW)	30–100	30.0011	67.6295	PG_104_	30–100	30.0026	32.5553	VG_85_	0.95–1.1	1.0606	1.0411
*PG*_*4*_	30–100	30.0020	30.0036	PG_105_	30–100	30.0014	52.2936	VG_87_	0.95–1.1	1.0738	1.0598
*PG*_*6*_	30–100	30.0007	30.0276	PG_107_	30–100	30.0018	57.7409	VG_89_	0.95–1.1	1.0718	1.0465
*PG*_*8*_	30–100	30.0085	30.0033	PG_110_	30–100	30.0022	30.0016	VG_90_	0.95–1.1	1.0562	1.0389
*PG*_*10*_	165–550	315.560	165.001	PG_111_	40.8–136	40.8005	40.8009	VG_91_	0.95–1.1	1.0613	1.0416
*PG*_*12*_	55.5–185	67.4081	135.879	PG_112_	30–100	30.0059	51.9958	VG_92_	0.95–1.1	1.0593	1.0379
*PG*_*15*_	30–100	30.0031	85.5395	PG_113_	30–100	30.0025	30.0034	VG_99_	0.95–1.1	1.0520	1.0388
*PG*_*18*_	30–100	30.0018	30.0776	PG_116_	30–100	30.0013	76.5788	VG_100_	0.95–1.1	1.0565	1.0389
*PG*_*19*_	30–100	30.0008	61.5287	VG_1_	0.95–1.1	1.0254	1.0076	VG_103_	0.95–1.1	1.0539	1.0403
*PG*_*24*_	30–100	30.0019	30.0011	VG_4_	0.95–1.1	1.0527	1.0228	VG_104_	0.95–1.1	1.0481	1.0378
*PG*_*25*_	96–320	152.388	96.0010	VG_6_	0.95–1.1	1.0457	1.0193	VG_105_	0.95–1.1	1.0466	1.0377
*PG*_*26*_	124.2–414	220.928	124.201	VG_8_	0.95–1.1	1.0393	1.0383	VG_107_	0.95–1.1	1.0394	1.0376
*PG*_*27*_	30–100	30.0002	49.7993	VG_10_	0.95–1.1	1.0494	1.0443	VG_110_	0.95–1.1	1.0491	1.0418
*PG*_*31*_	32.1–107	32.1000	61.0029	VG_12_	0.95–1.1	1.0395	1.0185	VG_111_	0.95–1.1	1.0584	1.0507
*PG*_*32*_	30–100	30.0013	39.5734	VG_15_	0.95–1.1	1.0403	1.0260	VG_112_	0.95–1.1	1.0397	1.0376
*PG*_*34*_	30–100	30.0031	65.0953	VG_18_	0.95–1.1	1.0426	1.0269	VG_113_	0.95–1.1	1.0510	1.0333
*PG*_*36*_	30–100	30.0012	54.3823	VG_19_	0.95–1.1	1.0402	1.0266	VG_116_	0.95–1.1	1.0603	1.0389
*PG*_*40*_	30–100	30.0019	99.9967	VG_24_	0.95–1.1	1.0597	1.0434	QC_5_	0–25	24.8405	17.2361
*PG*_*42*_	30–100	30.0018	100	VG_25_	0.95–1.1	1.0724	1.0518	QC_34_	0–25	0.01739	0.00708
*PG*_*46*_	35.7–119	35.7003	82.1696	VG_26_	0.95–1.1	1.0792	1.0586	QC_37_	0–25	0.02634	0.00288
*PG*_*49*_	91.2–304	161.452	142.111	VG_27_	0.95–1.1	1.0478	1.0342	QC_44_	0–25	4.38078	4.73039
*PG*_*54*_	44.4–148	44.4003	147.912	VG_31_	0.95–1.1	1.0426	1.0315	QC_45_	0–25	18.7874	19.0624
*PG*_*55*_	30–100	30.0098	72.4298	VG_32_	0.95–1.1	1.0464	1.0330	QC_46_	0–25	23.5131	22.2057
*PG*_*56*_	30–100	30.0020	99.9780	VG_34_	0.95–1.1	1.0489	1.0297	QC_48_	0–25	8.09067	7.49906
*PG*_*59*_	76.5–255	124.379	250.749	VG_36_	0.95–1.1	1.0457	1.0272	QC_74_	0–25	24.9614	22.7474
*PG*_*61*_	78–260	122.965	78.0026	VG_40_	0.95–1.1	1.0315	1.0263	QC_79_	0–25	24.9937	24.9996
*PG*_*62*_	30–100	30.0002	65.8296	VG_42_	0.95–1.1	1.0325	1.0265	QC_82_	0–25	24.8981	24.9971
*PG*_*65*_	147.3–491	288.992	147.302	VG_46_	0.95–1.1	1.0472	1.0308	QC_83_	0–25	11.6755	11.2571
*PG*_*66*_	147.6–492	288.832	147.603	VG_49_	0.95–1.1	1.0580	1.0292	QC_105_	0–25	21.4127	24.3775
*PG*_*70*_	30–100	30.0013	30.0003	VG_54_	0.95–1.1	1.0350	1.0264	QC_107_	0–25	24.3334	17.2322
*PG*_*72*_	30–100	30.0007	30.0008	VG_55_	0.95–1.1	1.0352	1.0262	QC_110_	0–25	25	24.9554
*PG*_*73*_	30–100	30.0008	30.0005	VG_56_	0.95–1.1	1.0350	1.0260	T_8_	0.9–1.1	0.98613	1.01559
*PG*_*74*_	30–100	30.0031	97.3429	VG_59_	0.95–1.1	1.0558	1.0260	T_32_	0.9–1.1	1.05612	1.06118
*PG*_*76*_	30–100	30.0011	99.9864	VG_61_	0.95–1.1	1.0617	1.0264	T_36_	0.9–1.1	0.99155	1.00470
*PG*_*77*_	30–100	30.0044	99.9955	VG_62_	0.95–1.1	1.0582	1.0253	T_51_	0.9–1.1	0.97685	0.99911
*PG*_*80*_	173.1–577	347.822	286.897	VG_65_	0.95–1.1	1.0636	1.0406	T_93_	0.9–1.1	0.98627	1.00866
*PG*_*85*_	30–100	30.0012	30.2624	VG_66_	0.95–1.1	1.0719	1.0317	T_95_	0.9–1.1	0.99915	1.00478
*PG*_*87*_	31.2–104	31.2000	31.2005	VG_69_	0.95–1.1	1.0631	1.0349	T_102_	0.9–1.1	0.98323	0.98364
*PG*_*89*_	212.1–707	384.629	212.100	VG_70_	0.95–1.1	1.0473	1.0376	T_107_	0.9–1.1	0.95142	0.97568
*PG*_*90*_	30–100	30.0000	99.9790	VG_72_	0.95–1.1	1.0582	1.0458	T_127_	0.9–1.1	0.99570	0.98415
*PG*_*91*_	30–100	30.0006	30.0075	VG_73_	0.95–1.1	1.0528	1.0425	Fuel cost ($/h)		134,943.8	155,624.1
*PG*_*92*_	30–100	30.0004	30.0069	VG_74_	0.95–1.1	1.0346	1.0363	Ploss (MW)		58.20613	16.79906
*PG*_*99*_	30–100	30.0017	39.2696	VG_76_	0.95–1.1	1.0157	1.0242	VD (p.u)		2.704451	1.689343
*PG*_*100*_	105.6–352	177.426	105.786	VG_77_	0.95–1.1	1.0407	1.0344	L-index		0.062471	1.689343
*PG*_*103*_	42–140	42.0014	42.0009	VG_80_	0.95–1.1	1.0480	1.0399	PG_69_ (MW) [0–805.5]		371.1412	2.156425

**Figure 24:**
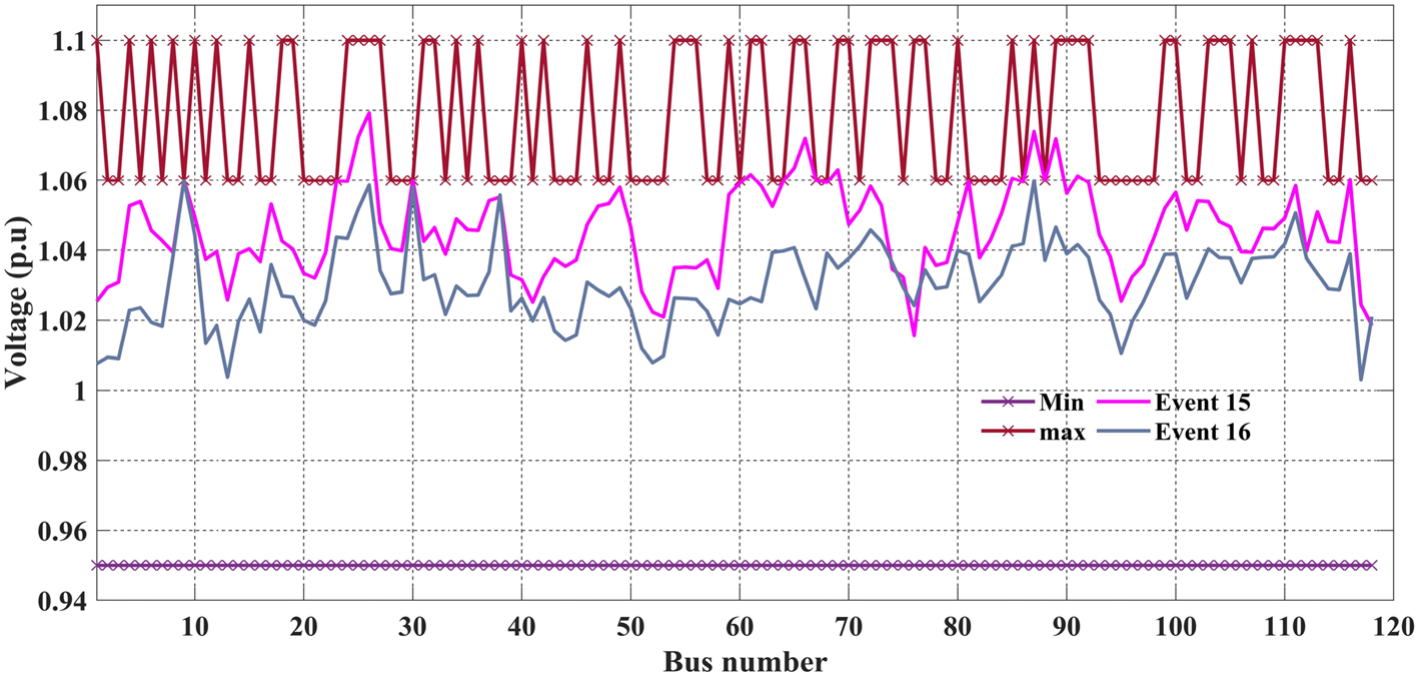
Event-15 and 16: Voltage profile of C2oDE-FR-ECM For IEEE 118-bus systems.

**Figure 25:**
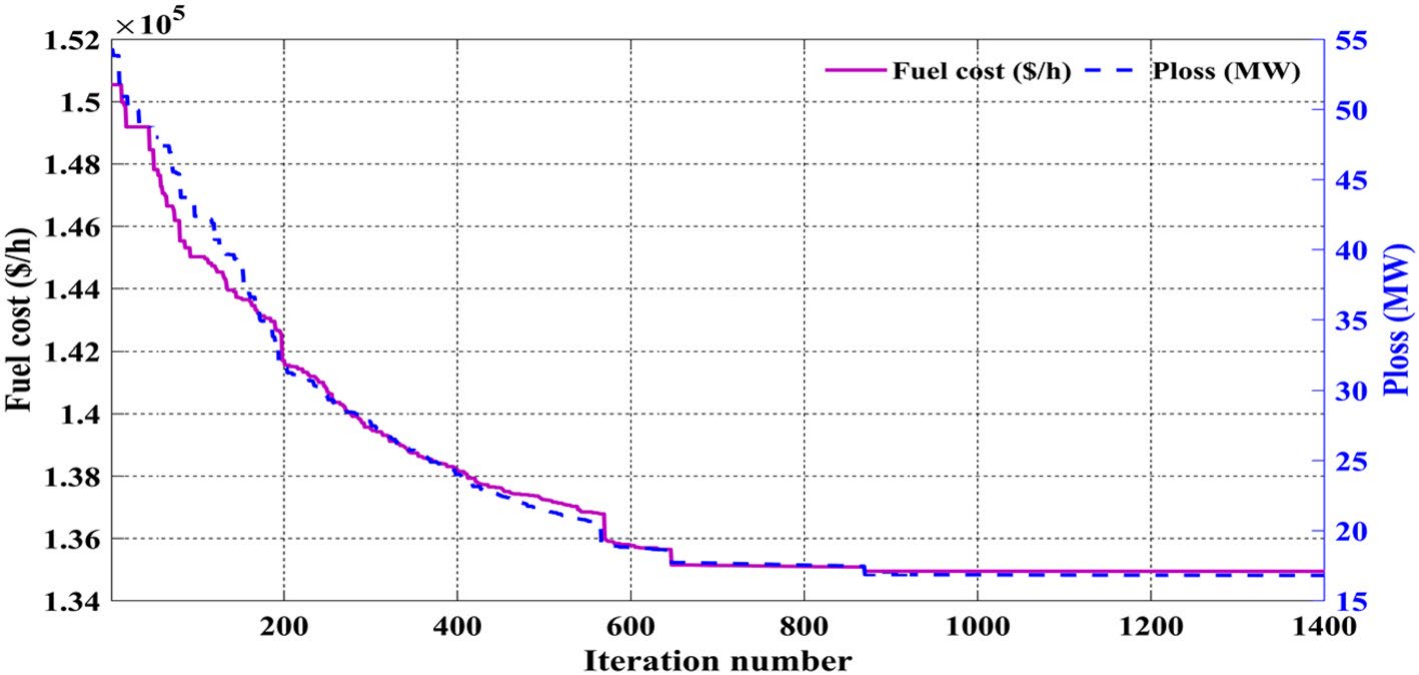
Convergence curves of event-15 and 16 (C2oDE-FR-ECM) for IEEE 118-bus.

Allowable values of MW and MVAr rating of generators, the voltage level of transformers, and the MVAr rating of shunt VAR compensators are taken from^26^, and Table 14 clearly shows that in events 15 and 16, all the control variables are fully satisfied the minimum and maximum limit. The results of event 15 and event 16 are that the basic fuel cost is 134,943.8 $/h and active power losses are 16.79906 MW, respectively. Figure 24 shows the voltage profile of all the buses and the minimum and maximum limits, while Fig. 25 gives the convergence curves of events 15 and 16.

However, Table 15 shows the comparative results of the proposed C2oDE-FR-ECM with the recently implemented DE variants in the literature. Table 15 shows that the proposed algorithm finds a better approximate optimal solution than all the other state-of-the-art evolutionary algorithms.

**Table 15:** Comparison of proposed algorithms with the past studies of IEEE 118-bus system.

Event #	Algorithm	Fuel Cost ($/h)	Ploss (MW)
Event 15	FR-ECM	134,943.8	58.20613
	ECM-DE^6^	135,055.7	60.9596
	DE^30^	143,169.2	60.5
	IMODE^31^	135,443.2	67.8
	SHADE^32^	135,386.9	56.4
	ABC^33^	151,132.5	97.0
Event 16	FR-ECM	155,624.1	16.7
	ECM-DE^6^	155,724.9	17.6
	DE^30^	155,999.0	36.8
	IMODE^31^	155,041.5	21.0
	SHADE^32^	156,165.2	18.3
	ABC^33^	155,809.4	73.3

## Conclusion

Optimal power flow (OPF) is a highly complex, constrained, and non-linear problem in a power system. In the solution of OPF problems without using suitable CHTs, the decision variable of the system may be violated and given poor safety, ill-functioning protective devices, and unnecessary power losses, especially with a static penalty function. Therefore, during the operation of the power system, constraint handling techniques (CHT) are responsible for optimizing objective functions subject to decision variables, and constraint functions should be within safe limits. Therefore, the application and usefulness of two CHTs, such as feasibility rule (FR) and ε constraint method (ECM), and their combinations with outstanding global optimizer C2oDE (C2oDE-FR, C2oDE-ECM, C2oDE-FR-ECM, C2oDE-ECM-FR) have been presented and used to solve OPF problem taking into various non-linear constraints. Three standard test networks, small to large-scale power system networks such as IEEE 30, 57, and 118-bus, are scrutinized to solve OPF problems with the CHTs group that helps achieve the best feasible solution in most of the events. A comparative analysis of the four techniques reveals the challenge of definitively establishing the superiority of one CHT over others in various OPF events. However, combining CHTs such as C2oDE-FR-ECM and C2oDE-ECM-FR method demonstrates considerable efficacy in achieving nearly optimal solutions in most events. However, it does not guarantee the most optimal solution or rapid convergence in all events.

Nonetheless, the significance of an efficient constraint-handling technique cannot be overstated. As our study demonstrates, inadequate CHT, mainly the penalty approach, may unknowingly lead to violations of network parameter limits. Therefore, to ensure a feasible solution to the OPF problem, the power system constraints must be within defined limits, is essential for its secure and proper functioning. The recommended configurations of CHT effectively bring the network to the desired state compared with several other methods in the past study.

## Data Availability

The data of proposed standard IEEE test systems used to support the findings of this study have been found in the open-source MTPOWER Package^26^. The datasets used and/or analyzed during the current study available from the corresponding author on reasonable request**.**
